# Biological expressions of early life trauma in the immune system of older adults

**DOI:** 10.1371/journal.pone.0286141

**Published:** 2023-06-21

**Authors:** Grace A. Noppert, Kate A. Duchowny, Rebecca Stebbins, Allison E. Aiello, Jennifer B. Dowd, Philippa Clarke

**Affiliations:** 1 Institute for Social Research, University of Michigan, Ann Arbor, Michigan; 2 Department of Epidemiology and Biostatistics, University of California, San Francisco, California, United States of America; 3 Social, Genetic, & Developmental Psychiatry Centre, Institute of Psychiatry, Psychology, and Neuroscience, King’s College London, London, United Kingdom; 4 Carolina Population Center, University of North Carolina at Chapel Hill, Chapel Hill, North Carolina, United States of America; 5 Department of Epidemiology, University of North Carolina at Chapel Hill, Chapel Hill, North Carolina, United States of America; 6 Leverhulme Centre for Demographic Science, University of Oxford, Oxford, United Kingdom; Montana State University, UNITED STATES

## Abstract

**Background:**

Poor immune function is associated with increased risk for a number of age-related diseases, however, little is known about the impact of early life trauma on immune function in late-life.

**Methods:**

Using nationally representative data from the Health and Retirement Study (n = 5,823), we examined the association between experiencing parental/caregiver death or separation before age 16 and four indicators of immune function in late-life: C-reactive Protein (CRP), Interleukin-6 (IL-6), soluble Tumor Necrosis Factor (sTNFR), and Immunoglobulin G (IgG) response to cytomegalovirus (CMV). We also examined racial/ethnic differences.

**Findings:**

Individuals that identified as racial/ethnic minorities were more likely to experience parental/caregiver loss and parental separation in early life compared to Non-Hispanic Whites, and had poorer immune function in late-life. We found consistent associations between experiencing parental/caregiver loss and separation and poor immune function measured by CMV IgG levels and IL-6 across all racial/ethnic subgroups. For example, among Non-Hispanic Blacks, those that experienced parental/caregiver death before age 16 had a 26% increase in CMV IgG antibodies in late-life (β = 1.26; 95% CI: 1.17, 1.34) compared to a 3% increase in CMV antibodies among Non-Hispanic Whites (β = 1.03; 95% CI: 0.99, 1.07) controlling for age, gender, and parental education.

**Interpretation:**

Our results suggest a durable association between experiencing early life trauma and immune health in late-life, and that structural forces may shape the ways in which these relationships unfold over the life course.

## Introduction

Early life experiences of stress and trauma have been repeatedly shown to have profound implications for health throughout adulthood including increased risk for obesity, physical inactivity, smoking, diabetes, cardiovascular disease, and poor mental health [1–3]. Yet, to date, few studies have investigated the effects of early life trauma on the immune system, particularly patterns of immune system aging. While there is a substantial body of work examining associations between stress and trauma on singular biomarkers of inflammation (e.g., CRP and IL-6) [4], an incomplete picture remains since prior research has almost exclusively focused on broad indicators of immune function that lack the specificity needed to understand the mechanisms underlying the stress-immunity relationship. Therefore, examining additional markers of immune system aging may shed light on the ways that the immune system ages in response to stress and trauma with implications for clinical responses to early life stress and trauma.

Childhood experiences of stress and trauma (e.g., adverse childhood events (ACEs)) have been shown to have profound implications for health throughout adulthood including increased risk for obesity, physical inactivity, smoking, diabetes, cardiovascular disease, and poor mental health.[1–3, 5, 6] While the cumulative experience of trauma such as what is captured in the ACEs score is certainly relevant for understanding the ensuing health consequences, it may also be that there are unique associations depending on the specific type of trauma experienced [7, 8]. Examining how traumatic events experienced during childhood offers a unique opportunity in understanding how stressful life events become embodied and contribute to immune system aging [7, 9]. Parental/caregiver death, for example, is often an acute, large-scale stressor that is experienced with little or no warning or plausible explanation, that has a cascade of life course implications for household resource availability, disruption of daily life, and elevated physiological stress responses [10]. Parental separation is a distinct form of trauma as it threatens the attachment between parent/caregiver and child with long-term consequences for physical, emotional, immune response, and cognitive development for years to come [8].

Early evidence suggests that experiences of childhood trauma characterized by various forms of familial abuse, neglect, parental loss, and household/familial trauma are increasing as a result of the COVID-19 pandemic [11–14]. A study using national data from the Centers for Disease Control and Prevention from 2013–2017 found that 3.5% of children in the U.S. experienced parental death before the age of 18 [15]. However, current estimates suggest that more than 116,000 children will experience parental loss due to COVID-19 specifically, a 17 to 20% increase in parental loss compared to annual rates prior to COVID-19 [16, 17]. Moreover, the rise in traumatic experiences is not distributed equally: children and adolescents of low socioeconomic status (SES) and minority race/ethnicity status are more likely to have experienced economic hardships, have had a family member die from COVID-19, [17] or have had educational disruptions as a result of the pandemic [18–20].

Traumatic events may best be conceptualized as part of the larger constellation of the social determinants of health. Therefore, understanding the importance of specific traumatic events in early childhood for later life health is of critical public health importance, especially as it relates to health inequities throughout the life course. Yet, there are unanswered questions with regard to what biological systems are most vulnerable to the effects of early life trauma and its long-term health sequalae. Prior research indicates that exposure to stress may elicit chronic activation of the immune system leading to declines in immune function and a chronic induction of the inflammatory response [21, 22]. Chronically elevated levels of pro-inflammatory markers such as C-Reactive Protein (CRP) and IL-6 are associated with cardiovascular disease, [23] disability, [24] frailty, [25, 26] and mortality [27]. However, it is unknown whether immune system dysfunction may be rooted in experiences occurring in early life, and experiences of early life trauma in particular.

We examined the association between experiencing parental/caregiver loss or parental separation before the age of 16 years and four distinct measures of immune function among older adults using a nationally representative cohort of older U.S. adults. Each measure represents separate, but interrelated, aspects of immune function that vary both with age, and in response to social and environmental stressors, though not uniformly [28]. The findings contribute to our understanding of the potential implications of the rise childhood trauma on life course patterns of immune health [29].

## Material & methods

### Study population

The Health and Retirement Study (HRS) is an on-going, nationally representative longitudinal survey of adult Americans, which began in 1992 and includes over 20,000 adults above the age of 50 years. The HRS employs a multi-stage probability survey design of non-institutionalized, community dwelling Americans [30, 31]. The survey design also oversamples certain demographic groups. Follow-up occurs every two years and new cohorts are added to maintain the nationally representativeness of the survey. The main survey, or the core interview, occurs every two years. Beginning in 2006, the HRS began employing a mixed-mode design for follow-up interviews. For each follow-up wave, half of the sample receives the enhanced face-to-face interview which also involves physical and biological measurements, as well as an in-depth psychosocial questionnaire. The other half is interviewed over the phone. Each half sample alternates the mode of the interview such that the enhanced face-to-face is available for each individual every four years. There are also a series of supplementary studies conducted that provide valuable data for the current investigation.

Informed consent is obtained prior to each interview. Participants are provided a written informed consent document, are read a confidentiality statement, and then are asked to give oral consent.

We used immune function data from the 2016 Venous Blood Study (VBS). The VBS was an initiative added in 2016 for panel participants who completed the 2016 core interview. A trained phlebotomist came to the home of the participant and collected a venous blood draw [32]. Informed consent was also obtained for the VBS specifically.

Assessment of early life trauma was derived from the 2015/2017 life history mail survey (LHMS). The LHMS was delivered as a mail survey to respondents during the off-years of the main survey [33]. Participants may have completed the survey in 2015 or 2017. We used data from the LHMS to construct our measures of early life trauma.

Demographic data for the sample was drawn from the 2014–2016 core surveys and the RAND longitudinal file. RAND is a research organization that partners with the HRS to provide user-friendly summary variables based on the HRS surveys. These variables are made available to HRS users [34].

There were 9,920 participants that completed the life histories questionnaire, all of whom were eligible for the VBS. We then limited our sample to those that were eligible, consented, and completed the VBS. Our final analytic sample size included 5,823 participants who had complete immune function data and had non-zero survey weights. Full details of the sample construction and data collections procedures are provided in **S1 Fig**.

### Characterization of immune function in late-life

We used four blood serum measures of immune function in older age: immune response to cytomegalovirus (CMV) measured by the level of Immunoglobulin G (IgG) antibody, high sensitive C-reactive Protein (CRP), Interleukin-6 (IL-6), and soluble Tumor Necrosis Factor (sTNFR). All assays were performed at the University of Minnesota Advanced Research and Diagnostic Laboratory (ARDL) [32, 35]. CMV seroprevalence was measured using IgG antibodies to cytomegalovirus (CMV) in serum using the Roche e411 immunoassay analyzer. Results were reported both categorically (non-reactive, borderline, and reactive) and as continuous IgG antibodies. The lower limit of detection for the assay was 0.15 COI. Categorical results were non-reactive (<0.5 COI (cutoff interval), borderline (0.5 to < 1.0 COI), or reactive (≥ 1.0 COI). CRP was measured in serum using a latex-particle enhanced immunoturbidimetric assay kit. IL-6 was also measured in serum by a enzyme-linked immunosorbent assay (ELISA) technique. (soluble) Tumor Necrosis Factor (sTNFR-1) was measured in serum by an ELISA.

CRP, IL-6, and sTNFR are commonly used as an indicator of inflammatory dysregulation in social and behavioral research. CMV IgG response is an indicator of the immune system’s capacity to control a persistent viral infection, CMV. Higher levels of CMV IgG response may reflect an impaired immune response in which viral activity cannot be adequately controlled. Each continuous variable was log-transformed for statistical analyses.

### Experiences of parental/caregiver loss or parental separation in early life

We defined early life trauma with two key events: 1) loss of a parent or caregiver; and 2) separation from a parent for ≥6 months. During the LHMS, participants were asked to report on two separate questions regarding parental/caregiver loss and parental separation.

Participants were asked the following questions to determine loss of a parent or caregiver and received a score of 1 if they indicated yes and 0 if no:

- “Before the age of 16, did one or both of your biological or adoptive parents die?”

To determine parental separation, the following questions were asked:

- “Before the age of 16, were you ever separated from your mother (or father) for 6 months or longer?”

Participants who reported any of these experiences received a score of 1 for parental separation and those that experienced none of these experiences received a score of 0.

### Covariates

**Fig 1:**
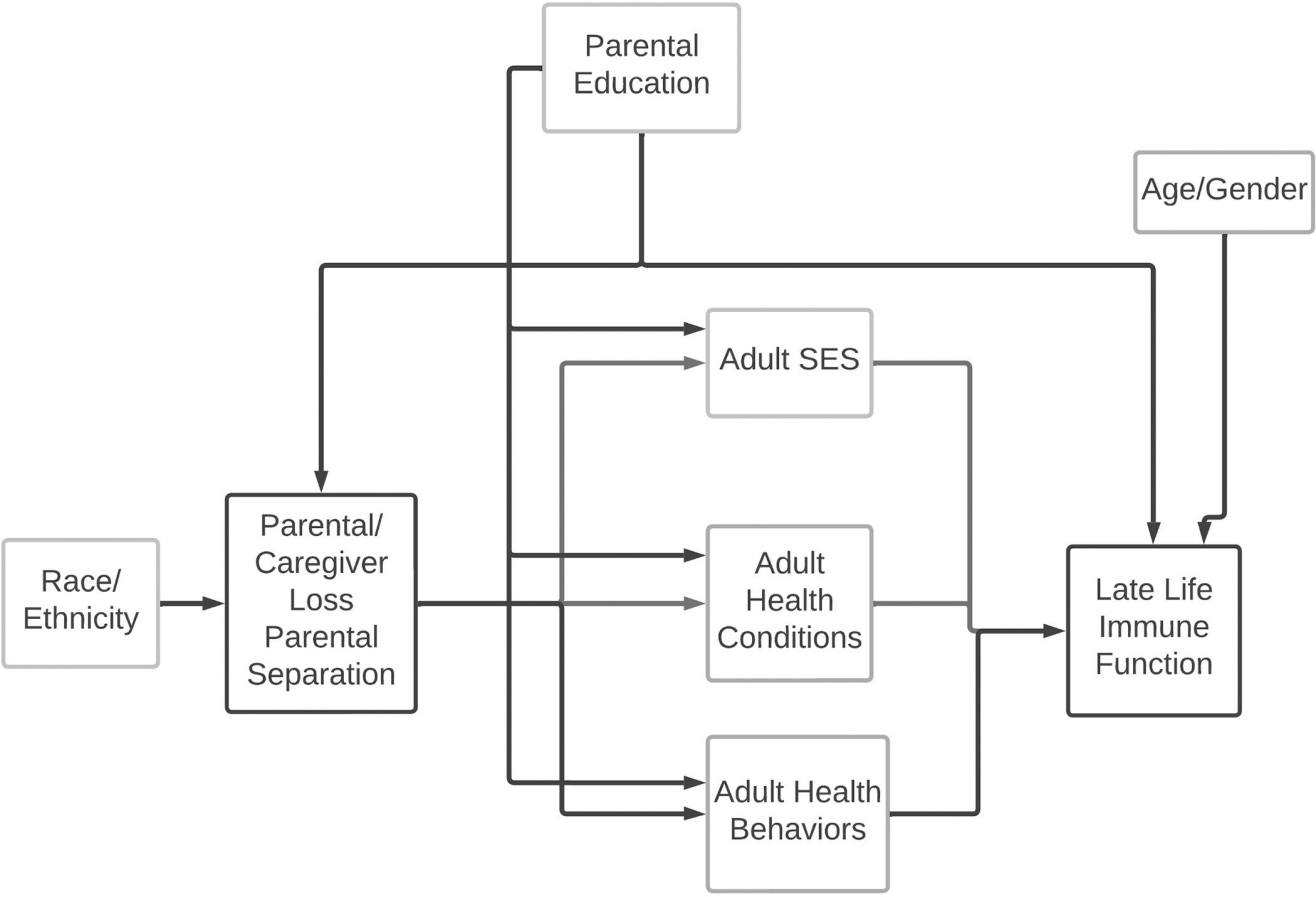
Conceptual diagram of the hypothesized relationship between early life trauma and late-life immune function. Note: not all of the hypothesized relationships between the variables are depicted below.

The following confounders not on the causal pathway were included in the analysis based on our conceptual diagram (**Fig 1**): age (modeled continuously in years), self-reported gender (woman/man), and parental education (≤8 years, 8–11 years, high school graduate, > high school).

We also investigated a series of potential health status and health behaviors mediators including smoking status, participant education, change in self-reported health, self-report of a change in health status, a chronic condition index, change in functional limitations, and body mass index (BMI). Additional details on each of these variables, including how they were derived, is given in **S1 File**.

### Assessment of racial and/or ethnic differences

Participants were asked to self-report both their race and whether they identified as Hispanic. We combined participant responses to a four-level race/ethnicity variable with the following categories: Non-Hispanic Black, Hispanic, Other race/ethnicity, and Non-Hispanic White.

We conceptualized race and or ethnicity as a proxy of one’s lived experience and a strong determinant of the burden of traumatic events experienced across the life course. Racial/ethnic categorizations are the result of the social process of racialization that has systematically placed certain individuals in separate and unequal contexts resulting in disproportionate exposures to social, environmental, and economic exposures. Thus, we follow the approach outlined by Ward et al. to assess the racial disparities including describing the distribution of both the exposure and outcome by racial/ethnicity [36].

### Analytical approach

Descriptive statistics were used to characterize the study population. We used multiple imputation techniques to account for the missing data (see **S2 File**). Linear regression models were used to estimate the association between experiences of parental/caregiver loss and parental separation and immune function. We modeled each of the four immune outcomes as log-transformed. The exponentiated effect estimates can then be interpreted as the percent change in the outcome associated with experiencing parental/caregiver loss and/or parental separation versus not.

We first developed a series of models in the full sample (see **S3 File, S1 and S2 Tables**). In the full sample models, Model 1 controlled for age and gender, Model 2 added parental education, Model 3 included an additional control for racial/ethnicity, and Model 4 included additional controls for adult SES, health behaviors, and health status indicators. We also estimated a model with two-way interaction terms between racial/ethnicity and both parental/caregiver loss and parental separation.

We then estimated racial/ethnicity-stratified models. In the racial/ethnicity-stratified models, Model 1 included controls for age and gender and Model 2 additionally controls for parental education. To further investigate potential mediators of the association between early life trauma and immune function, we also estimated an additional model (Model 3) controlling for indicators of health status and health behaviors. We note that this model (Model 3) and Model 4 in the full sample analyses may include mediators of the exposure-outcome relationship and may, therefore, represent over control. Thus, results should be interpreted with caution.

All analyses used the 2016 VBS survey weights and were conducted in R Version 1.4.1717.

### Sensitivity analyses

In sensitivity analyses, we also explored confounding by the state of birth of the respondent. There is evidence, particularly for CMV, of notable geographic variation in indicators of immunity [37]. Thus, we estimated an additional regression model controlling for age, gender, parental education, and state of respondent birth.

### Ethics approval

The current study is an analysis of secondary, de-identified data and was approved by the University of Michigan Institutional Review Board for the Health Sciences and Behavioral Sciences. The original HRS study received ethical approval, including obtaining informed consent and approval for biological specimen collection, from the IRB at the University of Michigan. Continued collection and production of HRS data comply with the requirements of the University of Michigan’s IRB.

## Results

### Descriptive findings

The sociodemographic profile of the full sample as well as the sample stratified by parental/caregiver loss and separation are provided in **Table 1**. Nineteen percent of participants reported experiencing parental/caregiver loss and 23% reported experiencing parental separation for ≥ 6 months before the age of 16. Nine percent of the sample reported experiencing both parental/caregiver loss and parental separation.

**Table 1:** Weighted demographic characteristics of the study sample in the Health and Retirement Study, n = 5,823. Results are displayed for the full sample and the sample stratified by both parental/caregiver death and parental separation.

	Full Sample		Stratified by Parental/Caregiver Death				Stratified by Parental Separation			
			No Parental/Caregiver Death (= 0)		Yes Parental/Caregiver Death (= 1)		No Parental Separation (= 0)		Yes Parental Separation (= 1)	
Variable	N	Mean (SE) or %	N	Mean (SE) or %	N	Mean (SE) or %	N	Mean (SE) or %	N	Mean (SE) or %
***Immune Measures***										
**Log-Transformed CMV IgG Antibody Level**	5823	3.50 (0.05)	4469	3.41 (0.05)	1186	3.92 (0.10)	4244	3.36 (0.06)	1491	3.97 (0.09)
*N Missing*		*0*	*0*		*0*		*0*		*0*	
**Log-Transformed CRP**	5823	0.83 (0.017)	4469	0.80 (0.019)	1186	0.94 (0.04)	4244	0.81 (0.02)	1491	0.89 (0.03)
*N Missing*		*0*	*0*		*0*		*0*		*0*	
**Log-Transformed sTNFR-1**	5823	7.42 (0.006)	4469	7.41 (0.007)	1186	7.48 (0.01)	4244	7.42 (0.007)	1491	7.44 (0.01)
*N Missing*		*0*	*0*		*0*		*0*		*0*	
**Log-Transformed IL-6**	5823	1.38 (0.16)	4469	1.13 (0.015)	1186	1.52 (0.03)	4244	1.36 (0.02)	1491	1.46 (0.03)
*N Missing*		*0*	*0*		*0*		*0*		*0*	
***Early-Life Trauma***										
**Experienced Parental/Caregiver Loss Before 16 Years**										
1 = Yes	1186	18.74					591	12.66	593	39.46
0 = No	4469	81.26					3602	87.34	861	60.54
*N Missing*		*168*					*51*		*37*	
**Experienced Parental Separation Before 16 Years**										
1 = Yes	1491	22.95	861	16.92	593	47.8				
0 = No	4244	77.05	3602	83.08	591	52.2				
*N Missing*		*88*	*6*		*2*					
***Demographics***										
**Age (years)**	5823	68.87 (0.016)	4469	68.50 (0.17)	1186	70.23 (0.37)	4244	68.62	1491	69.72 (0.34)
*N Missing*		*0*	*0*		*0*		*0*		*0*	
**Gender**										
Women	3452	55.79	2649	56.24	702	53.87	2519	56.31	881	53.84
Men	2371	44.21	1820	43.76	484	46.13	1725	43.69	610	46.16
*N Missing*		*0*								
**Race/Ethnicity**										
Non-Hispanic Black	964	8.55	610	6.75	325	16.07	538	6.06	410	16.62
Hispanic	309	3.71	201	3.28	96	5.51	203	3.26	103	5.33
Other Race	161	2.93	110	2.41	40	4.45	97	2.31	56	4.67
Non-Hispanic White	4385	84.82	3545	87.55	724	73.97	3404	88.37	920	73.38
*N Missing*		*4*	*3*		*1*		*2*		*2*	
**Parental Education**										
8 Years or Less of Education	586	9.16	418	8.27	150	13.35	460	9.55	121	7.99
9–11 Years of Education	588	10.82	471	10.65	101	11.5	454	11.19	124	8.99
High School Graduate	2021	45.74	1586	45.32	375	47.15	1469	44.19	519	51.49
Greater than High School	1427	34.28	1186	35.76	207	27.99	1093	35.07	313	31.52
***N Missing***		*1201*	*808*		*353*		*768*		*414*	
**Participant Education**										
Less than High School	215	3.01	132	2.36	76	5.94	144	2.71	68	4.05
High School Graduate	568	7.3	361	5.9	175	11.86	357	6.14	197	10.68
Some College	1896	31.71	1428	30.95	425	36.45	1384	31.23	488	34.03
College or Greater	3120	57.99	2533	60.79	502	45.74	2345	59.92	729	51.24
*N Missing*		*24*	*15*		*8*		*14*		*9*	
***Health Behaviors***										
**Smoking Status**										
Current Smoker	559	10.28	373	8.89	164	15.44	361	8.93	185	14.17
Former Smoker	2627	44.78	2013	44.44	546	46.23	1865	43.66	727	48.72
Never Smoker	2609	44.94	2061	46.67	473	38.32	2001	47.42	570	37.11
*N Missing*		*28*	*22*		*3*		*17*		*9*	
***Health Status Indicators***										
**Change in Self-Reported Health**	5816	0.06 (0.01)	4465	0.05 (0.014)	1184	0.09 (0.03)	4238	0.06 (0.01)	1490	0.05 (0.03)
***N Missing***		*7*	*4*		*2*		*6*		*1*	
**Self-Report of a Change in Overall Health Status**	5812	3.11 (0.009)	*4460*	3.10 (0.011)	1185	3.16 (0.02)	4234	3.11 (0.01)	1490	3.12 (0.02)
***N Missing***		*11*	*9*		*1*		*10*		*1*	
**Chronic Conditions Index**	5823	2.28 (0.02)	4469	2.21 (0.03)	1186	2.57 (0.06)	4244	2.19 (0.03)	1491	2.60 (0.05)
*N Missing*		*0*	*0*		*0*		*0*		*0*	
**Change in Functional Limitations**	5821	0.03 (0.01)	4467	0.04 (0.011)	1186	0.05 (0.03)	4242	0.04 (0.01)	1491	0.02 (0.02)
*N Missing*		*2*	*2*		*0*		*2*		*0*	
**Body Mass Index (BMI)**	5786	28.53 (0.10)	4440	28.46 (0.11)	1178	28.90 (0.23)	4216	28.44 (0.12)	1482	28.84 (0.20)
*N Missing*		*37*	*29*		*8*		*28*		*9*	

Those that reported experiencing parental/caregiver loss or parental separation had higher mean values of each of the four measured immune markers. Those that reported experiencing parental/caregiver loss had a mean value (standard error) of CMV IgG of 3.92 (0.10) compared to those that did not experience parental/caregiver loss with a mean value of 3.41 (0.05). Similarly, those that experienced parental/caregiver loss had a mean value of 0.94 (0.04) and 7.48 (0.01) for log-transformed CRP and sTNFR-1, respectively compared to 0.80 (0.019) and 7.41 (0.007) for those that did not experience parental/caregiver death. Finally, those that experienced parental/caregiver loss had a mean value of 1.52 (0.03) for IL-6 compared to a mean value of 1.13 (0.015) for those that did not experience parental/caregiver loss.

Among those that experienced parental separation, a similar pattern was observed. Those that experienced parental separation had mean (standard error) values of 3.97 (0.09), 0.89 (0.03), 7.44 (0.01), and 1.46 (0.03) for CMV IgG, CRP, sTNFR-1, and IL-6, respectively. This is compared to mean values of 3.36 (0.06), 0.81 (0.02), 7.42 (0.007), and 1.36 (0.02) for CMV IgG, CRP, sTNFR-1, and IL-6, respectively for those that did not experience parental separation.

### Racial/ethnic differences in immune function and early life trauma

As shown in **Table 2**, individuals who identified as racial/ethnic minorities had higher mean values of CMV IgG antibody levels, CRP, and IL-6 compared to non-Hispanic Whites (NHW). Moreover, individuals who identified as racial/ethnic minorities experienced more parental/caregiver loss and parental separation before age 16 than NHWs: 35% of non-Hispanic Blacks (NHB) experienced parent/caregiver loss compared to 28% of Hispanics, 30% of those classified as “Other” race and 16% of NHWs. A similar pattern was observed with parental separation.

**Table 2:** Contingency table showing the distribution of the immune measures and early life trauma experiences by racial/ethnicity.

	Non-Hispanic Black		Hispanic		Other Race/Ethnicity		Non-Hispanic White	
	N	Mean (SE) or %	N	Mean (SE) or %	N	Mean (SE) or %	N	Mean (SE) or %
***Immune Measures***								
**Log-Transformed CMV IgG Antibody Level**	964	4.99 (0.09)	309	4.96 (0.16)	161	4.77 (0.21)	4385	3.24 (0.05)
*N Missing*	*0*		*0*		*0*		*0*	
**Log-Transformed CRP**	964	1.13 (0.05)	309	0.95 (0.08)	161	0.81 (0.08)	4385	0.80 (0.02)
*N Missing*	*0*		*0*		*0*		*0*	
**Log-Transformed sTNFR-1**	964	7.42 (0.02)	309	7.43 (0.04)	161	7.35 (0.03)	4385	7.43 (0.006)
*N Missing*	*0*		*0*		*0*		*0*	
**Log-Transformed IL-6**	964	1.60 (0.03)	309	1.49 (0.07)	161	1.39 (0.09)	4385	1.36 (0.01)
*N Missing*	*0*		*0*		*0*		*0*	
***Early-Life Trauma***								
**Experienced Parental/Caregiver Loss Before 16 Years**								
1 = Yes	325	35.41	96	27.88	40	29.85	724	16.29
0 = No	610	64.59	201	72.12	110	70.15	3545	83.71
*N Missing*	*29*		*12*		*11*		*116*	
**Experienced Parental Separation Before 16 Years**								
1 = Yes	410	44.87	103	32.68	56	37.53	920	19.78
0 = No	538	55.13	203	67.32	97	62.47	3404	80.22
*N Missing*	*16*		*3*		*8*		*61*	

We formally tested for statistical interaction between racial/ethnicity and both parental/caregiver loss and parental separation by fitting models including two-way interaction terms. (See **S3 Table**). Based on the assessment of the statistical interactions, the differences in the distribution of both the exposure and outcomes by racial/ethnicity, and our *a priori* hypotheses regarding the structural influences of racial/ethnicity, we proceeded with racial/ethnicity-stratified models.

### Associations between parental/caregiver loss and immune function across racial/ethnic subgroups

**Fig 2:**
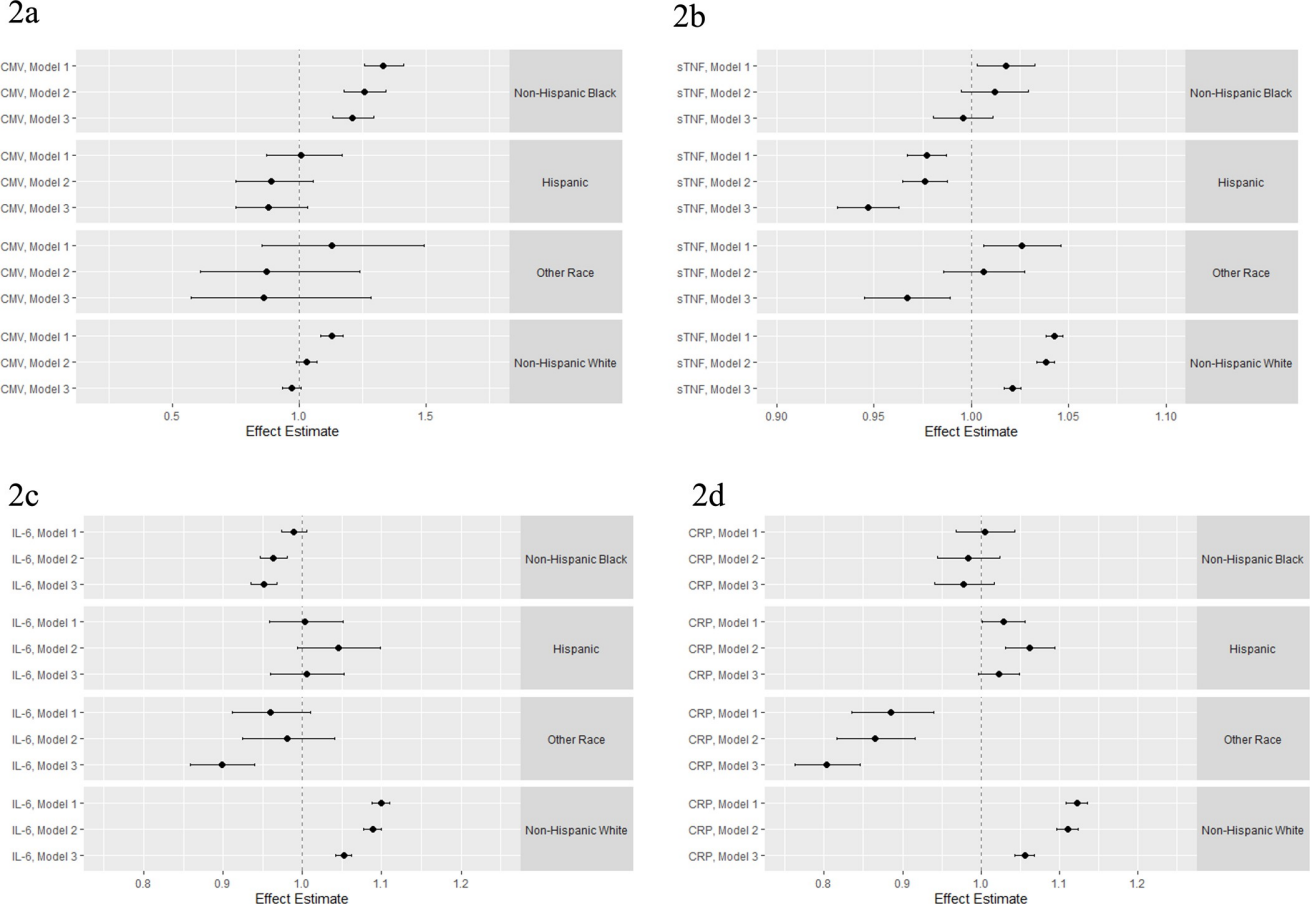
Results of the regression analysis estimating the association between experiencing parental/caregiver death before the age of 16 years and CMV (A), sTNF (B), IL-6 (C), and CRP (D) stratified by race/ethnicity. **Model 1** controls for age at the baseline interview in 2016 and gender. **Model 2** includes additional controls for parental education. **Model 3** includes additional controls for participant education, smoking status, change in self-reported health, self-report of a chance in health status, chronic conditions index, change in functional limitations, and BMI.

**Fig 2** shows the main effect estimates of the regression analyses estimating the association between experiencing parental/caregiver loss before the age of 16 and each immune measure in late-life, stratified by racial/ethnicity. For CMV IgG antibody levels, the strongest associations were among those who identified as NHB (**Fig 2A**) for whom experiencing the loss of a parent/caregiver was associated with a 26% increase in the CMV antibody level in late-life compared to those who did not experience parent/caregiver loss in a model controlling for age, gender, and parental education (Model 2, β = 1.26; 95% CI: 1.17, 1.34). Among those who identified as NHW, parent/caregiver loss was associated a 3% increase in CMV antibody level compared to those that did not experience parental/caregiver loss (Model 2; β = 1.03; 95% CI: 0.99, 1.07). Adding controls for adult SES, health behaviors, and health status indicators (Model 3) attenuated the observed estimates though notably the association for those who identified as NHB remained.

There was limited evidence of an association between parental/caregiver loss and sTNF levels in late-life for any racial/ethnic group (**Fig 2B**).

We observed an association between parental/caregiver loss and IL-6 levels in late-life but not across every racial/ethnic subgroup. Among both those who identified as NHW and Hispanic, experiencing parental/caregiver loss was associated with a 9% and 4% increase in IL-6 levels in late-life, respectively, in models controlling for age, gender, and parental education (Model 2; NHWs β = 1.09; 95% CI: 1.08, 1.10; Hispanic β = 1.04; 95% CI: 0.99, 1.10) (**Fig 2C**). In Model 3, we saw an attenuation of the estimates for all racial/ethnic subgroups except those who identified as NHB for whom the inclusion of control variables for adult SES, health behaviors, and health status indicators resulted in a larger estimate in the opposite direction (Model 3; 0.95; 95% CI: 0.93, 0.97). A similar pattern was observed for CRP (**Fig 2D**).

Full regression results are presented in **S4 Table.**

### Associations between parental separation and immune function across racial/ethnic subgroups

**Fig 3:**
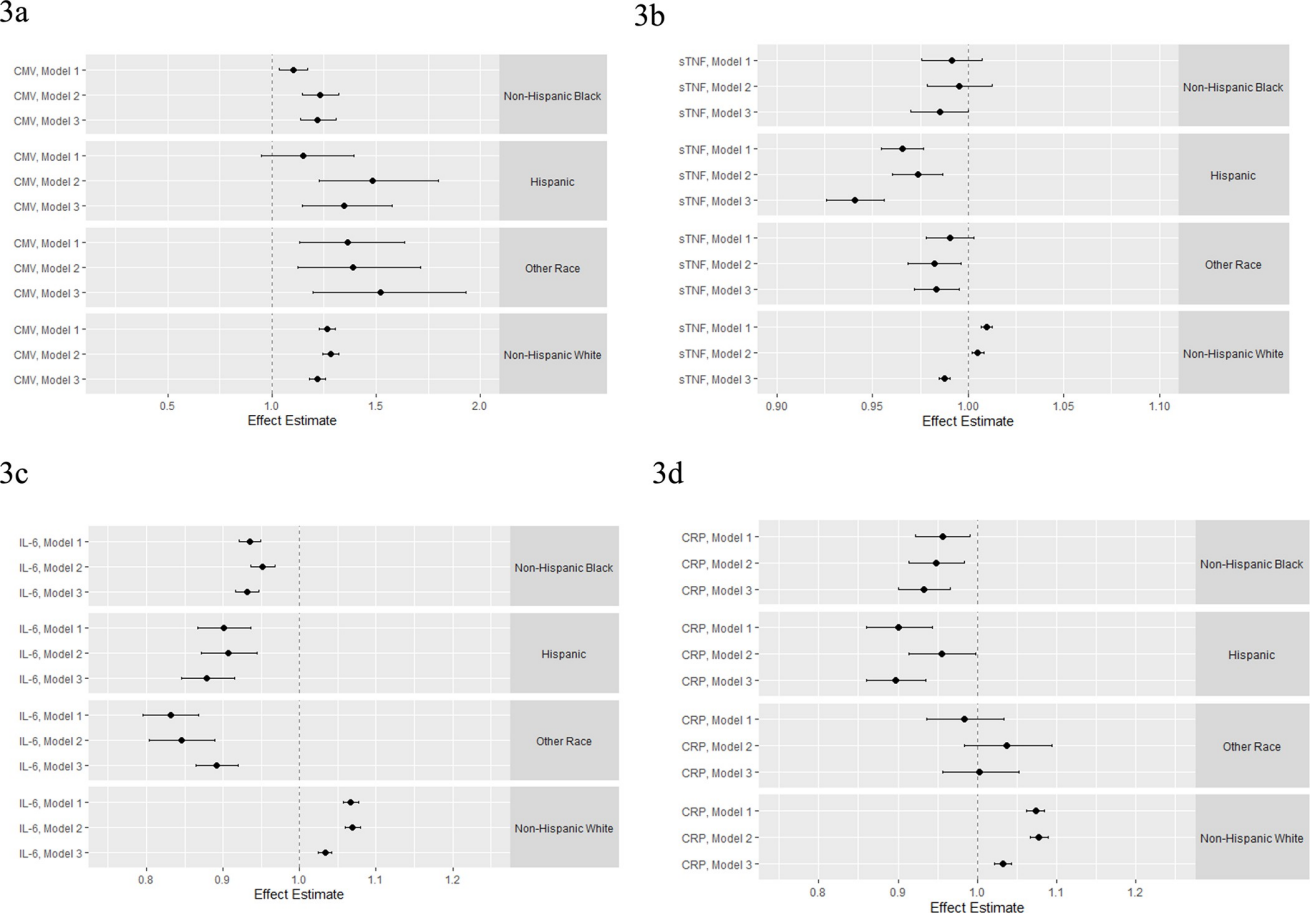
Results of the regression analysis estimating the association between experiencing parental separation before the age of 16 years and CMV (A), sTNF (B), IL-6 (C), and CRP (D) stratified by race/ethnicity. **Model 1 controls for age at the baseline interview in 2016 and gender. Model 2** includes additional controls for parental education. **Model 3** includes additional controls for participant education, smoking status, change in self-reported health, self-report of a chance in health status, chronic conditions index, change in functional limitations, and BMI.

**Fig 3** shows the main effects results of the regression analyses estimating the association between experiencing parental separation for 6 months or more before the age of 16 and each of the four immune measures in late-life stratified by racial/ethnicity. Among those who identified as Hispanic, experiencing parental separation was associated with a 48% increase in CMV antibody levels in a model controlling for age, gender, and parental education (Model 2; β = 1.48; 95% CI: 1.23, 1.80) (**Fig 3A**). A similar pattern was observed for the other racial/ethnic subgroups though the effect estimates were smaller. The estimates for all racial/ethnic subgroups were robust, though attenuated, to the inclusion of adult SES, health behaviors, and health status indicators (Model 3). For example, the association among those who identified as Hispanic in Model 3 was attenuated to β = 1.35 (95% CI: 1.15, 1.58).

We found limited evidence for an association between experiencing parental separation and sTNF levels in late-life (**Fig 3B**).

There was evidence of an association between experiencing parental separation and mean IL-6 levels across racial/ethnic groups (**Fig 3C**). However, with the exception of the those who identified as NHW, the associations were in the opposite direction. For example among those who identified as Hispanic, experiencing parental separation was associated with a 9% reduction in mean IL-6 levels in late-life (Model 2 β = 0.91; 95% CI: 0.87, 0.94). Among those who identified as both NHB and Hispanic, the inclusion of adult SES, health behaviors, and health status indicators resulted in a stronger association. In contrast, among those who identified as NHW experiencing parental separation was associated with a 7% increase in mean IL-6 levels in late-life (Model 2 β = 1.07 95% CI: 1.06, 1.08). This association was further attenuated in Model 3 (β = 1.03; 95% CI: 1.02, 1.04). Again, the patterns for CRP were similar to that observed for IL-6 (**Fig 3D**).

Full results are presented in the **S5 Table**. Additionally, the results from the full sample analyses are also included in see **S1 and S2 Tables.**

### Sensitivity analyses

In sensitivity analyses, we estimated an additional model controlling for age, gender, parental education, and state of respondent birth. Controlling for state of birth resulted in an attenuation of the effect estimates, most notably with regards to the association with CMV IgG levels for those who identified as NHB and NHW (see **S6 Table**). Future work should further investigate the role of early life conditions, including the neighborhood environment in the pathway from early life trauma and late-life immune function.

## Discussion

Using a nationally representative sample of older adults with retrospective data on early life trauma, we found associations between experiences of parental/caregiver loss and parental separation before the age of 16, and indicators of poor immune function in late-life. There were three main findings from this study. First, consistent with prior studies, we found that neither experiences of early life trauma nor immune function were distributed equitably. The inequities in the distribution of the exposures and outcomes speak to the pervasive effects of structural racism, which, through myriad mediating mechanisms, places racialized minority populations at greater risk of childhood traumas and can have long-term implications for immune health. Second, the association between parental/caregiver loss and separation and immune function varied according to the specific immune outcome investigated and this relationship differed across racial/ethnic subgroups, highlighting the complex biological and social processes underlying these relationships. Third, CMV appears to be particularly sensitive to experiences of early life trauma. Previous studies have relied heavily on singular biomarkers of inflammation without interrogating other markers of immune function that may indicate system-wide dysfunction such as immune response to cytomegalovirus (CMV).

Our findings add to the growing body of literature documenting the sustained impact of the early life environment on health throughout the life course [2, 38]. We extend prior work in this area by also showing how the trauma-immune aging relationship differs by racial/ethnic subgroups and by examining multiple indicators of immune function. Consistent with previous studies, we found mixed results with regards to the association between early life trauma and inflammatory markers. More work is needed to understand why we observed reduced levels of IL-6 and CRP for those who identified as NHB and Hispanic that experienced early life trauma [4, 39]. The consistent associations we observed with CMV IgG levels suggest that immune response to persistent viral infections may be a particularly salient indicator of overall immune fitness that is responsive to social stressors as has been documented in other studies [40–42]

There are several plausible biologic mechanisms by which traumatic events experienced in early life get “under the skin” to influence immune function in older age. One proposed mechanism is the conserved transcriptional response to adversity (CTRA) in leukocytes [43]. The CTRA is hypothesized to be a mammalian adaptive immune response to chronic stress and/or traumatic events and characterized by an upregulation of pro-inflammatory genes and a simultaneous down-regulation of antiviral activities. In addition, emerging evidence points to changes in gene expression induced by stress and trauma early in life that become embedded in the molecular architecture of an individual [43]. This may partially explain why we see a pattern of high inflammation and reduced immune control of CMV (i.e., reduced antiviral activity) among adults that can be linked to their reports of trauma in childhood.

Alternatively, it possible that individuals who experience more trauma in early life are also more likely to experience socioeconomic disadvantage across multiple domains. Children experiencing more socioeconomic disadvantage may be more likely to be exposed to CMV, exposed earlier in the life course, and experience re-exposure to CMV all of which may result in higher CMV IgG titers across the life course, including in late-life [44, 45]. Future studies are needed to discern the timing of exposure to CMV, the initial dose amount of the primary exposure, and how often re-exposure occurs to capture the dynamic process of CMV across the life course [44]. Furthermore, exposure to continued disadvantage and/or chronic stress across the life course can have long-term consequences for immune health through other pathways not mediated by CMV. Thus, studies that include multiple measures of immune health measured at multiple points in the life course are needed to elucidate these pathways.

There are several limitations that must be noted. First, this study is only nationally representative of the U.S. population and thus generalizations to other populations should be made with caution. Additionally, assessment of childhood trauma is based on participants self-report of experiences decades prior. This may result in differential recall bias. However, a 2016 study compared a prospective ACE measure to a retrospective ACE measure in the same cohort and both were correlated to subjective and objective adult health outcomes [46]. They found that the retrospective ACE measure underestimated the true effect of adversity on objective measures of health. Given these findings, we hypothesize that our results are likely an underestimate of the true magnitude of the association between childhood trauma and immune function. Further, the ways in which these childhood traumas are experienced as stressful is likely differential based on the individual. Future studies should investigate not only the occurrence of childhood traumas but the level of stress these traumas induced.

Additionally, the immune measures were only captured in late-life which poses a potential issue of selection bias. It may be that individuals exposed to childhood trauma and/or those of more advanced immune age may be less likely to survive long enough to be enrolled in this cohort. Moreover, while we observed consistent associations with CMV, immune control of CMV is a dynamic process occurring across the life course and is heavily influenced by the initial infectious dose as well as re-exposure to the virus, a process we could not disentangle with the available data.

Nonetheless, there are several strengths to our study. First, we focus on the critical window of childhood, an understudied exposure period in the immune function literature. Our findings shed light on the importance of examining the role of negative childhood experiences that are often overlooked when focusing exclusively in mid- and late-life periods. This is especially important since our results, along with others, suggest childhood trauma may place individuals on a trajectory of poor health including poor immune health.(6) Second, we leverage multiple immune measures with retrospective data on early life trauma in a nationally representative and diverse cohort of middle and older adults.

## Conclusion

Our study suggests that parental/caregiver loss and separation may be expressed in immune health in late-life. These results are set against the backdrop of an acute and unprecedented rise in childhood trauma from COVID-19, specifically related to the loss and disruption of caregiving, underscoring both the public health and clinical importance of these findings [29].

## Supporting information

S1 File Derivation of study sample and variable measurement. (PDF)

S2 File Missing data. (PDF)

S3 File Full sample analyses. (PDF)

S1 Fig Sample composition. Sample flow chart showing how the study sample was derived, n = 5,823.
(PDF)

S1 Table Exponentiated regression coefficients estimating the association between experiencing parental/caregiver loss before the age of 16 years and CMV, sTNFR, IL-6, and CRP. (PDF)

S2 Table Exponentiated regression coefficients estimating the association between experiencing parental separation before the age of 16 years and CMV, sTNFR, IL-6, and CRP. (PDF)

S3 Table Results of the regression analysis assessing two-way interactions between both experiencing parental/caregiver loss and race/ethnicity; and experiencing parental separation and race/ethnicity for four immune health indicators. (PDF)

S4 Table Exponentiated regression coefficients estimating the association between experiencing parental/caregiver loss before the age of 16 years and CMV (Panel A), sTNFR (Panel B), IL-6 (Panel C), and CRP (Panel D) stratified by race/ethnicity.
(PDF)

S5 Table Exponentiated regression coefficients estimating the association between experiencing parental separation before the age of 16 years and CMV (Panel A), sTNFR (Panel B), IL-6 (Panel C), and CRP (Panel D) stratified by race/ethnicity.
(PDF)

S6 Table Exponentiated regression coefficients estimating the association between experiencing parental/caregiver loss and parental separation before the age of 16 years and CMV, sTNF, IL-6, and CRP stratified by race/ethnicity. (PDF)
