# Supplementary material for: Biological expressions of early life trauma in the immune system of older adults
Source: PLoS One. 2023 Jun 21;18(6):e0286141. doi: 10.1371/journal.pone.0286141 (PMC10284407; doi:10.1371/journal.pone.0286141)
Supplement: S1 File — (PDF) [file pone.0286141.s001.pdf]

S1. File

A. Derivation of the Study Sample

Data collection consisted of face-to-face baseline interviews and primarily telephone interviews for follow-up waves, until 2006, at which point half of the sample was randomly assigned face-to-face interviews to enhance physical and biological measures. The half receiving face-to-face interviews alternated at each subsequent wave.[1] This study includes data on immune function from the 2016 Venous Blood Sample (VBS), which had a consent rate of 78.5%,[2] and on early life trauma from the 2006-2012 Leave-behind surveys and the 2015 and 2017 Life Histories Questionnaires.

Of the 29,541 survey participants who completed a core interview from 2006-2012, our analytic sample includes a subset of participants who completed the 2014 or 2016 core interviews, had complete life histories data, and participated in the VBS. Participants were ineligible if they were deceased, not cohort eligible, dropped out of the study, or not in the 2014 or 2016 wave, living in a nursing home, or had a proxy respondent. Our final analytic sample size included 6,176 participants who had complete outcome data on immune function and had non-zero survey weights. Full details of the sample construction are provided in **Supplementary Figure 1**.

B. Health Status, Adult Socioeconomic Status, and Health Behavior Variable Measurement

We included a series of variables indicative of the respondent's adult health, socioeconomic status, health behaviors, and health status indicators. These variables were derived from the RAND longitudinal file and we used the 2016 indicators. Additional information is available at the following site: https://www.rand.org/well-being/social-and-behavioral-policy/centers/aging/dataprod.html

Participant Education: This variable indicates years of education achieved by the respondent.

Smoking Status: A composite variable indicating whether the participants has ever smoked cigarettes, currently smokes, or has never smoked.

Change in Self-Reported Health: A measure of change in self-report of health status from the previous interview. Positive values indicate declines in self-rated health.

Self-Report of Health Change: A self-reported measure of how the respondent's health has changed since the previous interview. Higher values indicate deteriorating health.

Chronic Condition Index: This is the sum of indicators for whether a doctor has ever told the respondent that they have one of eight conditions: high blood pressure, diabetes, cancer, lung disease, stroke, psychiatric problems, and arthritis.

Change in Functional Limitations: A measure indicating the change in the index of activities of daily living from the previous interview. Higher values indicate declines in function (i.e., increased functional limitations).

Body Mass Index (BMI): A measure calculated by taking weight divided by the square of height. Height was converted into meters and weight into kilograms.

References

1. Juster FT, Suzman R. An overview of the Health and Retirement Study. *Journal of Human Resources*. 1995:S7-S56.
2. Crimmins EF, Jessica; Thyagarajan, Bharat; Weir, David. *Venous blood collection and assay protocol in the 2016 Health and Retirement Study*. 2017. *HRS Documentation Report*.
