# Supplementary material for: Biological expressions of early life trauma in the immune system of older adults
Source: PLoS One. 2023 Jun 21;18(6):e0286141. doi: 10.1371/journal.pone.0286141 (PMC10284407; doi:10.1371/journal.pone.0286141)
Supplement: S2 File — (PDF) [file pone.0286141.s002.pdf]

S2. File

Missing Data

We assessed patterns of missing data in our analytic sample (n = 5,823). Twenty-four percent of the population were missing data on one of more variables of interest. Most notably, 3% were missing data on parental death and 2% on parental separation and 21% were missing data on parental education.

We used multivariate imputation by chained equation (MICE) to impute the missing values for parental education, participant education, body mass index (BMI) smoking status, parental separation, and parental death.[3] We used 100 imputations to optimize precision in our estimates. We also used the following auxiliary variables to further optimize estimation in the imputation model: parental education, participant education, race/ethnicity, gender, age at baseline, and smoking status.
