# Supplementary material for: Biological expressions of early life trauma in the immune system of older adults
Source: PLoS One. 2023 Jun 21;18(6):e0286141. doi: 10.1371/journal.pone.0286141 (PMC10284407; doi:10.1371/journal.pone.0286141)
Supplement: S3 File — (PDF) [file pone.0286141.s003.pdf]

S3. File

Section 2: Analyses of the Full Sample

Association between experiencing early life parental/caregiver loss or parental separation and immune function

Overall, we found that experiencing parental/caregiver loss before age 16 was associated with all four immune measures in the full sample, though the strength of the association varied (**Supplementary Table 1**). For example, experiencing parental/caregiver loss was associated with a 48% increase in CMV antibody levels in late-life (95% CI1.45, 1.52) in a model controlling for age and gender (Model 1). The association remained when controlling for adult SES, health behaviors, and health status indicators (Model 4; 1.25; 95% CI 1.21, 1.28). For CRP, we found that experiencing parental loss was associated with a 11% increase in CMV antibody levels in late life (95% CI 1.10, 1.12) in a model controlling for age and gender (Model 1). The association remained in Model 4, though the strength of the association was attenuated (Model 4; 1.04; 95% CI 1.03, 1.05). There were also associations observed for sTNFr and IL-6.

We also observed associations between experiencing parental separation and CMV, IL-6 and CRP (**Supplementary Table 2**). Again, the strongest associations were observed for CMV. Experiencing parental separation was associated with 48% increase in CMV antibody levels in late life (95% CI: 1.45, 1.51) in a model controlling for age and gender (Model 1). Notably, this association was robust to the inclusion of controls for adult SES, health behaviors, and health status indicators (Model 4; 1.24; 95% CI 1.21, 1.26
