# Supplementary figures and images for: Biological expressions of early life trauma in the immune system of older adults
Source: PLoS One. 2023 Jun 21;18(6):e0286141. doi: 10.1371/journal.pone.0286141 (PMC10284407; doi:10.1371/journal.pone.0286141)

**S1 Figure.** Sample flow chart showing how the study sample was derived, n = 5,823.

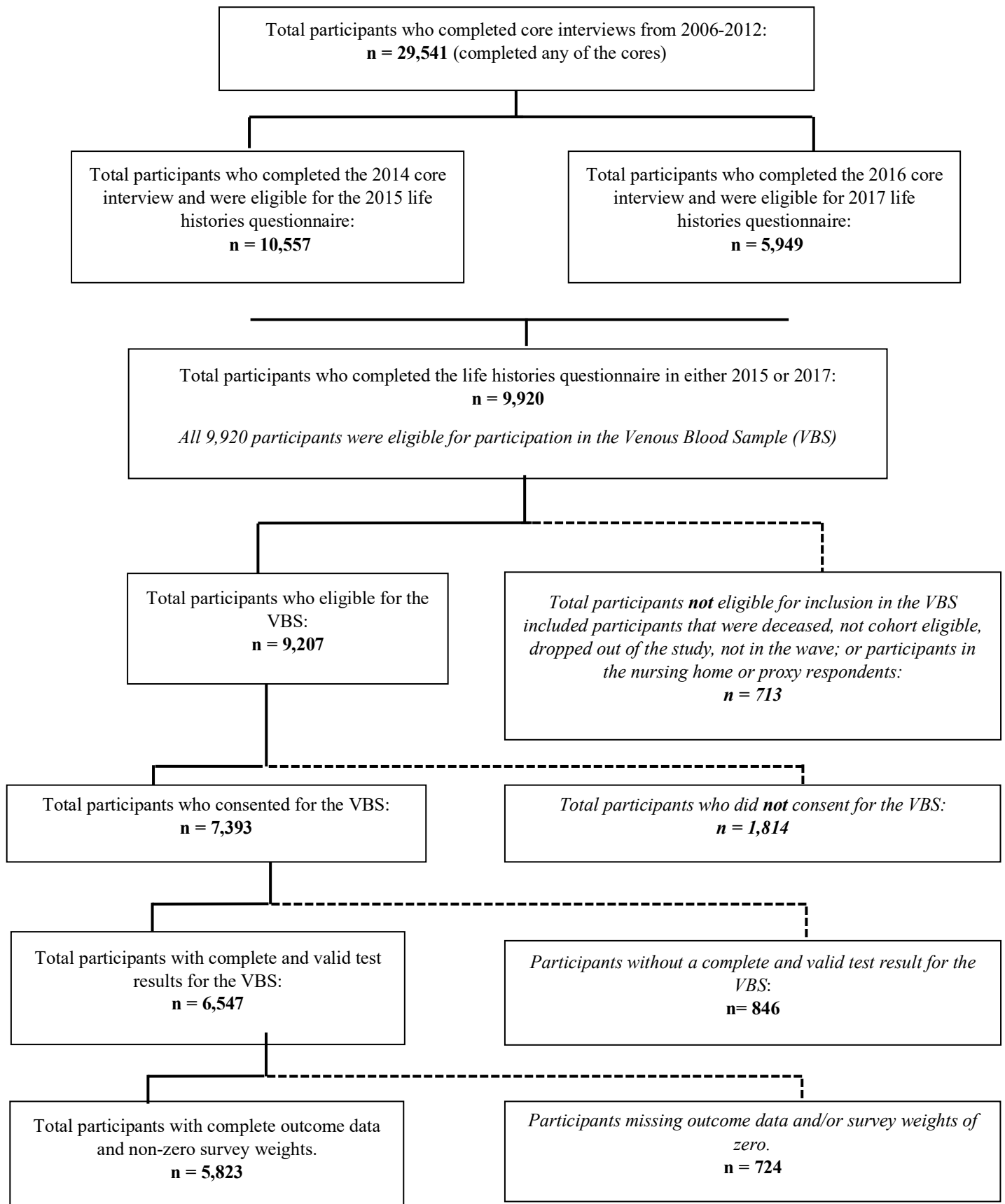

Supplement: S1 Fig — Sample flow chart showing how the study sample was derived, n = 5,823. (PDF) [file pone.0286141.s004.pdf]
