# Supplementary material for: Biological expressions of early life trauma in the immune system of older adults
Source: PLoS One. 2023 Jun 21;18(6):e0286141. doi: 10.1371/journal.pone.0286141 (PMC10284407; doi:10.1371/journal.pone.0286141)
Supplement: S1 Table — (PDF) [file pone.0286141.s005.pdf]

S1 Table. Exponentiated regression coefficients estimating the association between experiencing parental/caregiver loss before the age of 16 years and CMV, sTNFR, IL-6, and CRP. **Model 1** controls for age at the baseline interview in 2016 and gender. **Model 2** includes additional controls for parental education. **Model 3** includes additional controls for race/ethnicity. **Model 4** includes additional controls for participant education, smoking status, change in self-reported health, self-report of a chance in health status, chronic conditions index, change in functional limitations, and BMI.

	CMV								sTNFR							
	Model 1		Model 2		Model 3		Model 4		Model 1		Model 2		Model 3		Model 4	
	Estimate	CI	Estimate	CI	Estimate	CI	Estimate	CI	Estimate	CI	Estimate	CI	Estimate	CI	Estimate	CI
**Intercept**	0.59	0.45,0.77	4.98	3.5,7.08	1.92	1.31,2.81	2.92	1.35,6.33	618.42	615.49,621.37	684.20	680.21,688.21	693.20	689.1,697.32	485.30	479.57,491.1
**Experienced Parental/Caregiver Loss**	1.34	1.3,1.39	1.14	1.11,1.18	1.03	1.,1.06	0.98	0.95,1.01	1.03	1.03,1.04	1.03	1.02,1.03	1.03	1.0263,1.0345	1.01	1.01,1.02
**Age** (Years)	1.04	1.04,1.04	1.03	1.03,1.03	1.035	1.0347,1.035	1.04	1.03,1.04	1.0147	1.0146,1.0147	1.01	1.014,1.0141	1.014	1.0139,1.014	1.012	1.012,1.013
**Gender (Male vs Female)**	2.19	2.15,2.23	2.04	2.01,2.08	2.00	1.97,2.03	2.02	1.98,2.05	1.004	1.0036,1.0043	1.00	0.9991,0.9997	0.9994	0.99903,0.9997	0.995	0.995,0.996
**Parental Education** (Higher Values = Higher Education)			0.58	0.58,0.58	0.65	0.64,0.65	0.68	0.68,0.69			0.97	0.9696,0.9701	0.97	0.968,0.97	0.99	0.991,0.992
**Race/Ethnicity:** NH-Black					4.35	4.19,4.51	3.96	3.82,4.12					0.99	0.987,0.99	0.94	0.941,0.945
Hispanic					3.30	3.08,3.53	3.24	3.03,3.47					1.00	0.99,1.	0.99	0.986,0.994
Other Race					4.19	3.77,4.66	4.25	3.81,4.74					0.94	0.94,0.94	0.93	0.93,0.94
**Participant Education** (Higher Values = Higher Education)							0.82	0.81,0.82							0.96	0.961,0.962
**Smoking Status**							1.13	1.11,1.14							1.00	1.001,1.002
**Change in Self-Reported Health**							0.99	0.98,0.99							0.99	0.994,0.995
**Self-Report of Health Change**							0.89	0.88,0.9							1.02	1.02,1.021
**Chronic Condition Index**							1.03	1.03,1.04							1.056	1.055,1.056
**Change in Functional Limitations**							0.86	0.85,0.87							1.04	1.037,1.038
**BMI**							1.01	1.,1.01							1.01	1.011,1.012

	IL-6								CRP							
	Model 1		Model 2		Model 3		Model 4		Model 1		Model 2		Model 3		Model 4	
	Estimate	CI	Estimate	CI	Estimate	CI	Estimate	CI	Estimate	CI	Estimate	CI	Estimate	CI	Estimate	CI
**Intercept**	1.40	1.37,1.43	1.62	1.57,1.67	1.47	1.42,1.52	477.18	471.37,483.07	1.91	1.84,1.98	2.42	2.31,2.53	2.21	2.11,2.33	0.27	0.25,0.3
**Experienced Parental/Caregiver Loss**	1.09	1.09,1.1	1.0166	1.0166,1.0167	1.07	1.06,1.08	0.99	0.98,0.99	1.11	1.1,1.12	1.09	1.08,1.11	1.08	1.07,1.09	1.04	1.03,1.05
**Age** (Years)	1.017	1.017,1.0171	0.9520	0.9505,0.9535	1.02	1.02,1.02	1.01	1.0125,1.0126	0.999	0.9992,0.9993	0.9977	0.9977,0.9978	1.00	0.998,0.999	1.00	1.001,1.002
**Gender (Male vs Female)**	0.958	0.9566,0.9595	0.9406	0.94,0.9411	0.95	0.95,0.95	0.995	0.99,0.995	1.20	1.198,1.203	1.20	1.199,1.204	1.20	1.19,1.2	1.21	1.207,1.212
**Parental Education** (Higher Values = Higher Education)			0.9685	0.9684,0.9686	0.95	0.95,0.95	0.991	0.9912,0.9917			0.92	0.921,0.923	0.93	0.931,0.933	0.98	0.98,0.98
**Race/Ethnicity:** NH-Black					1.20	1.19,1.21	0.949	0.9468,0.9509					1.22	1.2,1.23	1.08	1.07,1.1
Hispanic					1.14	1.12,1.15	0.994	0.9905,0.9981					1.11	1.09,1.13	1.10	1.09,1.12
Other Race					1.06	1.04,1.09	0.940	0.9365,0.9435					0.98	0.963,1.003	1.01	0.99,1.03
**Participant Education** (Higher Values = Higher Education)							0.961	0.9604,0.9609							0.95	0.95,0.96
**Smoking Status**							1.004	1.003,1.004							1.12	1.121,1.125
**Change in Self-Reported Health**							0.995	0.9947,0.9951							0.99	0.99,0.992
**Self-Report of Health Change**							1.021	1.0203,1.021							1.06	1.06,1.065
**Chronic Condition Index**							1.056	1.0561,1.0566							1.06	1.055,1.057
**Change in Functional Limitations**							1.037	1.0362,1.0372							1.06	1.059,1.064
**BMI**							1.012	1.0114,1.0117							1.05	1.053,1.054
