# Supplementary material for: Biological expressions of early life trauma in the immune system of older adults
Source: PLoS One. 2023 Jun 21;18(6):e0286141. doi: 10.1371/journal.pone.0286141 (PMC10284407; doi:10.1371/journal.pone.0286141)
Supplement: S2 Table — (PDF) [file pone.0286141.s006.pdf]

S2 Table. Exponentiated regression coefficients estimating the association between experiencing parental separation before the age of 16 years and CMV, sTNFR, IL-6, and CRP. **Model 1** controls for age at the baseline interview in 2016 and gender. **Model 2** includes additional controls for parental education. **Model 3** includes additional controls for race/ethnicity. **Model 4** includes additional controls for participant education, smoking status, change in self-reported health, self-report of a chance in health status, chronic conditions index, change in functional limitations, and BMI.

	CMV								sTNFR							
	Model 1		Model 2		Model 3		Model 4		Model 1		Model 2		Model 3		Model 4	
	Estimate	CI	Estimate	CI	Estimate	CI	Estimate	CI	Estimate	CI	Estimate	CI	Estimate	CI	Estimate	CI
**Intercept**	0.60	0.46,0.77	5.68	4.02,8.02	2.25	1.55,3.28	3.44	1.6,7.41	605.72	603.12,608.33	675.26	671.34,679.2	682.07	678.04,686.13	477.18	471.37,483.07
**Experienced Parental/Caregiver Loss**	1.48	1.45,1.52	1.47	1.43,1.51	1.29	1.26,1.33	1.25	1.21,1.28	1.00	1.002,1.008	1.001	0.998,1.004	1.002	0.999,1.006	0.99	0.98,0.99
**Age** (Years)	1.04	1.0436,1.044	1.03	1.026,1.0264	1.03	1.03,1.03	1.03	1.034,1.035	1.0148	1.01479,1.01481	1.01	1.01412,1.01414	1.014	1.014,1.0141	1.01	1.0125,1.0126
**Gender (Male vs Female)**	2.20	2.16,2.23	2.05	2.01,2.09	2.01	1.98,2.04	2.02	1.99,2.06	1.003	1.003,1.004	0.999	0.9985,0.999	0.999	0.998,0.999	0.995	0.9947,0.9953
**Parental Education** (Higher Values = Higher Education)			0.58	0.57,0.58	0.65	0.64,0.65	0.68	0.678,0.685			0.97	0.968,0.969	0.968	0.968,0.968	0.991	0.9912,0.9917
**Race/Ethnicity:** NH-Black					4.03	3.88,4.18	3.70	3.56,3.84					0.994	0.992,0.995	0.95	0.9468,0.9509
Hispanic					3.16	2.95,3.38	3.11	2.9,3.32					0.9996	0.996,1.003	0.994	0.9905,0.9981
Other Race					3.94	3.55,4.38	4.00	3.59,4.46					0.941	0.939,0.943	0.94	0.9365,0.9435
**Participant Education** (Higher Values = Higher Education)							0.82	0.82,0.83							0.961	0.9604,0.9609
**Smoking Status**							1.11	1.1,1.12							1.0036	1.0032,1.004
**Change in Self-Reported Health**							0.99	0.98,0.99							0.995	0.9947,0.9951
**Self-Report of Health Change**							0.89	0.88,0.9							1.0206	1.0203,1.021
**Chronic Condition Index**							1.03	1.02,1.03							1.0563	1.0561,1.0566
**Change in Functional Limitations**							0.87	0.86,0.88							1.0367	1.0362,1.0372
**BMI**							1.01	1.004,1.007							1.012	1.0114,1.0117

	IL-6								CRP							
	Model 1		Model 2		Model 3		Model 4		Model 1		Model 2		Model 3		Model 4	
	Estimate	CI	Estimate	CI	Estimate	CI	Estimate	CI	Estimate	CI	Estimate	CI	Estimate	CI	Estimate	CI
**Intercept**	1.35	1.33,1.38	1.60	1.55,1.65	1.44	1.39,1.5	0.56	0.53,0.6	1.84	1.78,1.9	2.40	2.29,2.5	2.19	2.08,2.3	0.27	0.25,0.3
**Experienced Parental/Caregiver Loss**	1.05	1.04,1.06	1.05	1.04,1.06	1.04	1.03,1.04	1.01	0.998,1.013	1.07	1.06,1.08	1.07	1.06,1.08	1.05	1.04,1.06	1.014	1.01,1.02
**Age** (Years)	1.02	1.0172,1.0173	1.02	1.0167,1.0167[illegible]	1.02	1.017,1.018	1.02	1.018,1.018	0.9995	0.9994,0.9995	1.00	0.998,0.998	0.9985	0.998,0.999	1.0016	1.001,1.002
**Gender (Male vs Female)**	0.957	0.956,0.959	0.95	0.95,0.952	0.95	0.947,0.95	0.955	0.95,0.96	1.20	1.197,1.202	1.20	1.197,1.203	1.196	1.19,1.2	1.21	1.206,1.211
**Parental Education** (Higher Values = Higher Education)			0.94	0.937,0.938	0.95	0.949,0.95	0.987	0.987,0.988			0.92	0.918,0.919	0.93	0.929,0.93	0.98	0.977,0.978
**Race/Ethnicity:** NH-Black					1.20	1.19,1.21	1.108	1.1005,1.11597					1.22	1.201,1.235	1.09	1.07,1.1
Hispanic					1.14	1.12,1.15	1.113	1.1005,1.12532					1.115	1.095,1.136	1.11	1.09,1.12
Other Race					1.06	1.04,1.09	1.081	1.0611,1.10147					0.98	0.962,1.002	1.01	0.99,1.03
**Participant Education** (Higher Values = Higher Education)							0.94	0.943,0.945							0.95	0.95,0.953
**Smoking Status**							1.08	1.077,1.08							1.12	1.12,1.13
**Change in Self-Reported Health**							1.01	1.011,1.012							0.99	0.99,0.992
**Self-Report of Health Change**							1.00	1.003,1.006							1.06	1.06,1.065
**Chronic Condition Index**							1.07	1.065,1.066							1.06	1.055,1.057
**Change in Functional Limitations**							1.03	1.025,1.029							1.06	1.059,1.064
**BMI**							1.03	1.027,1.028							1.054	1.053,1.055
