# Supplementary material for: Biological expressions of early life trauma in the immune system of older adults
Source: PLoS One. 2023 Jun 21;18(6):e0286141. doi: 10.1371/journal.pone.0286141 (PMC10284407; doi:10.1371/journal.pone.0286141)
Supplement: S3 Table — (PDF) [file pone.0286141.s007.pdf]

Section 3: Interaction Analyses and Stratified Analyses

S3 Table. Results of the regression analysis assessing two-way interactions between both experiencing parental/caregiver loss and race/ethnicity; and experiencing parental separation and race/ethnicity for four immune health indicators.

	CMV<br>P Value	CRP<br>P Value	sTNF<br>P Value	IL-6<br>P Value
**Experiencing Parental/Caregiver Loss*Race Interactions**				
Experiencing Parental/Caregiver Loss*Non-Hispanic Black	0.40	0.16	0.44	0.02
Experiencing Parental/Caregiver Loss*Hispanic	0.50	0.95	0.14	0.84
Experiencing Parental/Caregiver Loss*Other Race	0.43	0.09	0.73	0.45
**Experiencing Parental Separation*Race Interactions**				
Experiencing Parental Separation*Non-Hispanic Black	0.99	0.10	0.80	0.03
Experiencing Parental Separation*Hispanic	0.53	0.37	0.66	0.08
Experiencing Parental Separation*Other Race	0.99	0.63	0.76	0.09
