# Supplementary material for: Biological expressions of early life trauma in the immune system of older adults
Source: PLoS One. 2023 Jun 21;18(6):e0286141. doi: 10.1371/journal.pone.0286141 (PMC10284407; doi:10.1371/journal.pone.0286141)
Supplement: S4 Table — Exponentiated regression coefficients estimating the association between experiencing parental/caregiver loss before the age of 16 years and CMV (Panel A), sTNFR (Panel B), IL-6 (Panel C), and CRP (Panel D) stratified by race/ethnicity. (PDF) [file pone.0286141.s008.pdf]

S4 Table. Exponentiated regression coefficients estimating the association between experiencing parental/caregiver loss before the age of 16 years and CMV (Panel A), sTNFR (Panel B), IL-6 (Panel C), and CRP (Panel D) stratified by race/ethnicity. **Model 1** controls for age at the baseline interview in 2016 and gender. **Model 2** includes additional controls for parental education. **Model 3** includes additional controls for participant education, smoking status, change in self-reported health, self-report of a chance in health status, chronic conditions index, change in functional limitations, and BMI.

Panel A. CMV

	Non-Hispanic Black CMV					
	Model 1		Model 2		Model 3	
	Est	CI	Est	CI	Est	CI
**Intercept**	21.26	7.64,59.2	99.17	21.09,466.26	220.74	19.56,2491.27
**Experienced Parental/Caregiver Loss**	1.33	1.26,1.41	1.26	1.17,1.34	1.21	1.13,1.29
**Age** (Years)	1.01	1.007,1.013	0.99	0.992,0.997	0.99	0.991,0.997
**Gender (Male vs Female)**	2.26	2.08,2.44	2.16	1.98,2.36	2.44	2.23,2.67
**Parental Education** (Higher Values = Higher Education)			0.72	0.69,0.74	0.71	0.69,0.73
**Participant Education** (Higher Values = Higher Education)					0.89	0.87,0.92
**Smoking Status**					1.12	1.09,1.16
**Change in Self-Reported Health**					1.00	0.98,1.02
**Self-Report of Health Change**					0.99	0.95,1.03
**Chronic Condition Index**					0.91	0.88,0.93
**Change in Functional Limitations**					1.09	1.06,1.11
**BMI**					0.98	0.98,0.99

	Hispanic CMV					
	Model 1		Model 2		Model 3	
	Est	CI	Est	CI	Est	CI
**Intercept**	2.56	0.04,153.14	10.05	0.15,690.54	403.77	1.21,135233.81
**Experienced Parental/Caregiver Loss**	1.01	0.87,1.17	0.89	0.75,1.05	0.88	0.75,1.03
**Age** (Years)	1.06	1.05,1.06	1.04	1.04,1.04	1.03	1.02,1.03
**Gender (Male vs Female)**	1.06	0.85,1.3	1.12	0.92,1.36	1.01	0.84,1.21
**Parental Education** (Higher Values = Higher Education)			0.62	0.58,0.66	0.68	0.63,0.73
**Participant Education** (Higher Values = Higher Education)					0.88	0.84,0.91
**Smoking Status**					0.68	0.59,0.79
**Change in Self-Reported Health**					0.89	0.83,0.94
**Self-Report of Health Change**					0.58	0.5,0.67
**Chronic Condition Index**					1.16	1.13,1.19
**Change in Functional Limitations**					0.82	0.77,0.87
**BMI**					0.82	0.77,0.87

	Other Race CMV					
	Model 1		Model 2		Model 3	
	Est	CI	Est	CI	Est	CI
**Intercept**	8.06	0.01,5051.07	10.48	0.,621056.12	1.71	0.,1254949545.22
**Experienced Parental/Caregiver Loss**	1.13	0.85,1.49	0.87	0.61,1.24	0.86	0.58,1.28
**Age** (Years)	1.04	1.03,1.04	1.04	1.03,1.04	1.03	1.026,1.035
**Gender (Male vs Female)**	1.18	0.84,1.64	1.16	0.78,1.74	1.26	0.83,1.92
**Parental Education** (Higher Values = Higher Education)			0.83	0.76,0.9	0.87	0.8,0.94
**Participant Education** (Higher Values = Higher Education)					1.15	1.01,1.31
**Smoking Status**					1.03	0.73,1.44
**Change in Self-Reported Health**					0.83	0.71,0.97
**Self-Report of Health Change**					1.70	1.29,2.24
**Chronic Condition Index**					1.07	1.02,1.13
**Change in Functional Limitations**					1.17	0.82,1.67
**BMI**					1.00	0.993,1.003

	Non-Hispanic White CMV					
	Model 1		Model 2		Model 3	
	Est	CI	Est	CI	Est	CI
**Intercept**	0.23	0.16,0.32	1.58	0.98,2.54	2.10	0.77,5.73
**Experienced Parental/Caregiver Loss**	1.13	1.09,1.17	1.03	0.99,1.07	0.97	0.93,1.01
**Age** (Years)	1.05	1.05,1.05	1.04	1.037,1.038	1.04	1.038,1.039
**Gender (Male vs Female)**	2.21	2.16,2.26	2.09	2.04,2.14	2.11	2.07,2.16
**Parental Education** (Higher Values = Higher Education)			0.63	0.63,0.64	0.68	0.67,0.68
**Participant Education** (Higher Values = Higher Education)					0.78	0.77,0.79
**Smoking Status**					1.14	1.13,1.16
**Change in Self-Reported Health**					1.00	0.99,1.01
**Self-Report of Health Change**					0.90	0.88,0.92
**Chronic Condition Index**					1.04	1.03,1.04
**Change in Functional Limitations**					0.82	0.81,0.84
**BMI**					1.01	1.008,1.011

Panel B. sTNFR

	Non-Hispanic Black sTNFR					
	Model 1		Model 2		Model 3	
	Est	CI	Est	CI	Est	CI
**Intercept**	673.81	637.44,712.26	678.66	605.48,760.68	471.02	413.14,537.02
**Experienced Parental/Caregiver Loss**	1.02	1.,1.03	1.01	0.99,1.03	1.00	0.98,1.01
**Age** (Years)	1.01	1.0129,1.0131	1.01	1.0139,1.0141	1.01	1.011,1.012
**Gender (Male vs Female)**	1.01	1.01,1.02	0.99	0.988,0.9976	0.97	0.97,0.98
**Parental Education** (Higher Values = Higher Education)			0.98	0.98,0.98	1.00	0.997,1.002
**Participant Education** (Higher Values = Higher Education)					0.98	0.98,0.98
**Smoking Status**					1.02	1.01,1.02
**Change in Self-Reported Health**					0.99	0.988,0.99
**Self-Report of Health Change**					1.03	1.02,1.03
**Chronic Condition Index**					1.08	1.079,1.082
**Change in Functional Limitations**					1.08	1.08,1.09
**BMI**					1.01	1.008,1.009

	Hispanic sTNFR					
	Model 1		Model 2		Model 3	
	Est	CI	Est	CI	Est	CI
**Intercept**	896.61	778.53,1032.61	926.66	731.26,1174.28	852.05	538.85,1347.31
**Experienced Parental/Caregiver Loss**	0.98	0.97,0.99	0.98	0.96,0.99	0.95	0.93,0.96
**Age** (Years)	1.01	1.01,1.0104	1.01	1.0098,1.0101	1.01	1.006,1.007
**Gender (Male vs Female)**	0.97	0.95,0.98	0.97	0.954,0.978	0.93	0.92,0.94
**Parental Education** (Higher Values = Higher Education)			0.98	0.97,0.98	1.01	1.01,1.02
**Participant Education** (Higher Values = Higher Education)					0.96	0.95,0.97
**Smoking Status**					1.06	1.06,1.07
**Change in Self-Reported Health**					0.94	0.94,0.95
**Self-Report of Health Change**					1.06	1.04,1.07
**Chronic Condition Index**					1.09	1.089,1.094
**Change in Functional Limitations**					1.04	1.04,1.05
**BMI**					1.00	1.001,1.002

	Other Race sTNFR					
	Model 1		Model 2		Model 3	
	Est	CI	Est	CI	Est	CI
**Intercept**	452.59	406.57,503.83	531.87	461.44,613.06	464.14	309.11,696.91
**Experienced Parental/Caregiver Loss**	1.03	1.01,1.05	1.01	0.99,1.03	0.97	0.95,0.99
**Age** (Years)	1.02	1.0175,1.0181	1.02	1.015,1.016	1.01	1.013,1.014
**Gender (Male vs Female)**	1.04	1.03,1.05	1.07	1.06,1.07	0.99	0.977,0.995
**Parental Education** (Higher Values = Higher Education)			0.96	0.95,0.96	0.99	0.99,0.996
**Participant Education** (Higher Values = Higher Education)					0.94	0.94,0.95
**Smoking Status**					0.94	0.93,0.94
**Change in Self-Reported Health**					1.00	0.995,1.002
**Self-Report of Health Change**					1.03	1.02,1.04
**Chronic Condition Index**					1.07	1.069,1.074
**Change in Functional Limitations**					1.09	1.08,1.1
**BMI**					1.01	1.007,1.011

	Non-Hispanic White sTNFR					
	Model 1		Model 2		Model 3	
	Est	CI	Est	CI	Est	CI
**Intercept**	611.73	608.43,615.05	688.88	684.69,693.11	465.42	459.37,471.56
**Experienced Parental/Caregiver Loss**	1.04	1.04,1.05	1.04	1.03,1.04	1.02	1.02,1.03
**Age** (Years)	1.01	1.01488,1.0149	1.01	1.0141,1.0141	1.01	1.01286,1.01293
**Gender (Male vs Female)**	1.00	1.004,1.005	1.00	0.9998,1.0006	1.00	1.001,1.002
**Parental Education** (Higher Values = Higher Education)			0.97	0.968,0.968	0.99	0.989,0.99
**Participant Education** (Higher Values = Higher Education)					0.96	0.961,0.962
**Smoking Status**					1.00	0.999,0.9997
**Change in Self-Reported Health**					1.00	0.999,0.9996
**Self-Report of Health Change**					1.02	1.018,1.0187
**Chronic Condition Index**					1.05	1.051,1.0517
**Change in Functional Limitations**					1.02	1.022,1.023
**BMI**					1.01	1.012,1.0128

Panel C. IL-6

	Non-Hispanic Black IL-6					
	Model 1		Model 2		Model 3	
	Est	CI	Est	CI	Est	CI
**Intercept**	1.37	1.16,1.62	1.11	0.88,1.39	0.47	0.31,0.72
**Experienced Parental/Caregiver Loss**	0.99	0.97,1.01	0.96	0.95,0.98	0.95	0.93,0.97
**Age** (Years)	1.01	1.01,1.02	1.02	1.02,1.02	1.02	1.02,1.02
**Gender (Male vs Female)**	1.17	1.15,1.19	1.15	1.13,1.17	1.13	1.11,1.15
**Parental Education** (Higher Values = Higher Education)			0.95	0.95,0.96	0.96	0.95,0.96
**Participant Education** (Higher Values = Higher Education)					1.03	1.03,1.04
**Smoking Status**					1.13	1.12,1.14
**Change in Self-Reported Health**					1.08	1.08,1.08
**Self-Report of Health Change**					0.97	0.96,0.98
**Chronic Condition Index**					1.04	1.03,1.04
**Change in Functional Limitations**					0.99	0.99,1.
**BMI**					1.02	1.01,1.02

	Hispanic IL-6					
	Model 1		Model 2		Model 3	
	Est	CI	Est	CI	Est	CI
**Intercept**	0.37	0.18,0.74	0.28	0.12,0.65	0.15	0.04,0.59
**Experienced Parental/Caregiver Loss**	1.00	0.96,1.05	1.04	0.99,1.1	1.01	0.96,1.05
**Age** (Years)	1.03	1.03,1.03	1.04	1.04,1.04	1.04	1.03,1.04
**Gender (Male vs Female)**	1.21	1.18,1.24	1.23	1.19,1.27	1.18	1.14,1.21
**Parental Education** (Higher Values = Higher Education)			1.04	1.03,1.05	1.08	1.07,1.09
**Participant Education** (Higher Values = Higher Education)					0.92	0.91,0.93
**Smoking Status**					1.09	1.06,1.13
**Change in Self-Reported Health**					1.03	1.02,1.05
**Self-Report of Health Change**					1.10	1.07,1.13
**Chronic Condition Index**					1.04	1.03,1.05
**Change in Functional Limitations**					1.05	1.04,1.07
**BMI**					1.02	1.02,1.02

	Other Race IL-6					
	Model 1		Model 2		Model 3	
	Est	CI	Est	CI	Est	CI
**Intercept**	1.30	0.69,2.45	2.41	1.11,5.24	0.59	0.11,3.12
**Experienced Parental/Caregiver Loss**	0.96	0.91,1.01	0.98	0.92,1.04	0.90	0.86,0.94
**Age** (Years)	1.01	1.01,1.02	1.01	1.01,1.01	1.00	1.,1.01
**Gender (Male vs Female)**	1.11	1.04,1.19	1.07	0.99,1.16	0.89	0.86,0.92
**Parental Education** (Higher Values = Higher Education)			0.94	0.93,0.96	1.11	1.07,1.14
**Participant Education** (Higher Values = Higher Education)					0.74	0.7,0.79
**Smoking Status**					0.87	0.84,0.9
**Change in Self-Reported Health**					0.87	0.83,0.9
**Self-Report of Health Change**					1.24	1.12,1.37
**Chronic Condition Index**					1.08	1.08,1.09
**Change in Functional Limitations**					1.04	1.,1.07
**BMI**					1.06	1.05,1.06

	Non-Hispanic White IL-6					
	Model 1		Model 2		Model 3	
	Est	CI	Est	CI	Est	CI
**Intercept**	1.43	1.39,1.47	1.66	1.59,1.73	0.65	0.6,0.7
**Experienced Parental/Caregiver Loss**	1.10	1.09,1.11	1.09	1.08,1.1	1.05	1.04,1.06
**Age** (Years)	1.02	1.02,1.02	1.02	1.02,1.02	1.02	1.02,1.02
**Gender (Male vs Female)**	0.92	0.92,0.92	0.92	0.92,0.92	0.93	0.93,0.93
**Parental Education** (Higher Values = Higher Education)			0.95	0.95,0.95	0.98	0.98,0.98
**Participant Education** (Higher Values = Higher Education)					0.95	0.95,0.95
**Smoking Status**					1.07	1.07,1.08
**Change in Self-Reported Health**					1.01	1.01,1.01
**Self-Report of Health Change**					0.99	0.99,1.
**Chronic Condition Index**					1.07	1.07,1.07
**Change in Functional Limitations**					1.04	1.04,1.04
**BMI**					1.03	1.03,1.03

Panel D. CRP

	Non-Hispanic Black CRP					
	Model 1		Model 2		Model 3	
	Est	CI	Est	CI	Est	CI
**Intercept**	1.66	1.01,2.73	2.06	1.15,3.69	0.23	0.09,0.58
**Experienced Parental/Caregiver Loss**	1.00	0.97,1.04	0.98	0.94,1.02	0.98	0.94,1.02
**Age** (Years)	1.00	0.999,1.001	1.00	).998,1.00001	1.01	1.005,1.007
**Gender (Male vs Female)**	1.44	1.4,1.47	1.45	1.41,1.49	1.33	1.3,1.37
**Parental Education** (Higher Values = Higher Education)			0.87	0.85,0.88	0.89	0.88,0.9
**Participant Education** (Higher Values = Higher Education)					1.02	1.01,1.03
**Smoking Status**					1.16	1.14,1.17
**Change in Self-Reported Health**					1.06	1.05,1.07
**Self-Report of Health Change**					0.97	0.95,0.99
**Chronic Condition Index**					1.08	1.08,1.09
**Change in Functional Limitations**					1.08	1.07,1.09
**BMI**					1.05	1.05,1.053

	Hispanic CRP					
	Model 1		Model 2		Model 3	
	Est	CI	Est	CI	Est	CI
**Intercept**	1.02	0.54,1.93	0.82	0.39,1.73	0.11	0.02,0.52
**Experienced Parental/Caregiver Loss**	1.03	1.001,1.056	1.06	1.03,1.09	1.02	0.997,1.049
**Age** (Years)	1.01	1.005,1.008	1.01	1.008,1.01	1.01	1.01,1.02
**Gender (Male vs Female)**	1.37	1.31,1.44	1.42	1.35,1.49	1.30	1.24,1.36
**Parental Education** (Higher Values = Higher Education)			1.05	1.02,1.07	1.10	1.08,1.13
**Participant Education** (Higher Values = Higher Education)					0.91	0.89,0.92
**Smoking Status**					1.09	1.06,1.13
**Change in Self-Reported Health**					1.02	1.01,1.03
**Self-Report of Health Change**					1.20	1.17,1.24
**Chronic Condition Index**					0.98	0.97,0.99
**Change in Functional Limitations**					1.13	1.12,1.14
**BMI**					1.05	1.045,1.049

	Other Race CRP					
	Model 1		Model 2		Model 3	
	Est	CI	Est	CI	Est	CI
**Intercept**	2.39	0.46,12.38	6.57	0.86,50.44	1.17	0.041,33.137
**Experienced Parental/Caregiver Loss**	0.89	0.83,0.94	0.86	0.82,0.92	0.80	0.763,0.846
**Age** (Years)	1.00	0.996,0.999	0.99	0.986,0.989	1.00	0.996,0.999
**Gender (Male vs Female)**	1.05	0.986,1.111	0.97	0.91,1.04	0.87	0.818,0.929
**Parental Education** (Higher Values = Higher Education)			0.85	0.84,0.86	0.89	0.877,0.901
**Participant Education** (Higher Values = Higher Education)					1.08	1.039,1.112
**Smoking Status**					0.98	0.945,1.009
**Change in Self-Reported Health**					1.23	1.202,1.257
**Self-Report of Health Change**					0.79	0.76,0.823
**Chronic Condition Index**					1.10	1.085,1.108
**Change in Functional Limitations**					1.32	1.26,1.38
**BMI**					1.05	1.05,1.06

	Non-Hispanic White CRP					
	Model 1		Model 2		Model 3	
	Est	CI	Est	CI	Est	CI
**Intercept**	1.91	1.83,1.99	2.33	2.2,2.47	0.28	0.25,0.32
**Experienced Parental/Caregiver Loss**	1.12	1.11,1.14	1.11	1.096,1.124	1.06	1.04,1.07
**Age** (Years)	1.00	0.999,1.	1.00	0.998,0.998	1.00	1.001,1.001
**Gender (Male vs Female)**	1.17	1.17,1.18	1.18	1.17,1.18	1.21	1.2,1.21
**Parental Education** (Higher Values = Higher Education)			0.94	0.94,0.94	0.99	0.98,0.99
**Participant Education** (Higher Values = Higher Education)					0.94	0.938,0.942
**Smoking Status**					1.12	1.118,1.123
**Change in Self-Reported Health**					0.97	0.97,0.98
**Self-Report of Health Change**					1.08	1.076,1.082
**Chronic Condition Index**					1.06	1.055,1.057
**Change in Functional Limitations**					1.05	1.046,1.051
**BMI**					1.05	1.054,1.055
