# Supplementary material for: Biological expressions of early life trauma in the immune system of older adults
Source: PLoS One. 2023 Jun 21;18(6):e0286141. doi: 10.1371/journal.pone.0286141 (PMC10284407; doi:10.1371/journal.pone.0286141)
Supplement: S5 Table — Exponentiated regression coefficients estimating the association between experiencing parental separation before the age of 16 years and CMV (Panel A), sTNFR (Panel B), IL-6 (Panel C), and CRP (Panel D) stratified by race/ethnicity. (PDF) [file pone.0286141.s009.pdf]

S5 Table. Exponentiated regression coefficients estimating the association between experiencing parental separation before the age of 16 years and CMV (Panel A), sTNFR (Panel B), IL-6 (Panel C), and CRP (Panel D) stratified by race/ethnicity. **Model 1** controls for age at the baseline interview in 2016 and gender. **Model 2** includes additional controls for parental education. **Model 3** includes additional controls for participant education, smoking status, change in self-reported health, self-report of a chance in health status, chronic conditions index, change in functional limitations and BMI.

Panel A. CMV

	Non-Hispanic Black CMV					
	Model 1		Model 2		Model 3	
	Est	CI	Est	CI	Est	CI
**Intercept**	17.92	6.73,47.7	91.21	20.18,412.16	223.77	20.3,2466.82
**Experienced Parental Separation**	1.10	1.04,1.17	1.23	1.15,1.32	1.22	1.14,1.31
**Age** (Years)	1.01	1.01,1.02	1.00	0.99,1.	0.995	0.992,0.998
**Gender (Male vs Female)**	2.24	2.06,2.42	2.13	1.95,2.32	2.41	2.21,2.64
**Parental Education** (Higher Values = Higher Education)			0.71	0.68,0.73	0.70	0.68,0.73
**Participant Education** (Higher Values = Higher Education)					0.88	0.86,0.91
**Smoking Status**					1.12	1.08,1.16
**Change in Self-Reported Health**					0.99	0.97,1.02
**Self-Report of Health Change**					0.98	0.94,1.03
**Chronic Condition Index**					0.91	0.88,0.94
**Change in Functional Limitations**					1.09	1.07,1.11
**BMI**					0.98	0.98,0.98

	Hispanic CMV					
	Model 1		Model 2		Model 3	
	Est	CI	Est	CI	Est	CI
**Intercept**	2.43	0.04,137.31	11.85	0.22,628.93	414.54	1.71,100363.37
**Experienced Parental Separation**	1.15	0.95,1.39	1.48	1.23,1.8	1.35	1.15,1.58
**Age** (Years)	1.06	1.06,1.06	1.04	1.04,1.05	1.03	1.02,1.03
**Gender (Male vs Female)**	1.08	0.86,1.35	1.16	0.95,1.41	1.04	0.87,1.25
**Parental Education** (Higher Values = Higher Education)			0.60	0.57,0.64	0.66	0.61,0.7
**Participant Education** (Higher Values = Higher Education)					0.91	0.87,0.94
**Smoking Status**					0.66	0.58,0.75
**Change in Self-Reported Health**					0.88	0.82,0.95
**Self-Report of Health Change**					0.61	0.53,0.7
**Chronic Condition Index**					1.14	1.11,1.18
**Change in Functional Limitations**					0.80	0.75,0.86
**BMI**					0.98	0.97,0.98

	Other Race CMV					
	Model 1		Model 2		Model 3	
	Est	CI	Est	CI	Est	CI
**Intercept**	8.08	0.02,2866.37	15.23	0.,562612.85	3.06	0, > 10.0
**Experienced Parental Separation**	1.36	1.13,1.64	1.39	1.13,1.71	1.52	1.2,1.93
**Age** (Years)	1.04	1.03,1.04	1.04	1.03,1.04	1.03	1.02,1.03
**Gender (Male vs Female)**	1.14	0.81,1.59	1.06	0.71,1.58	1.14	0.75,1.72
**Parental Education** (Higher Values = Higher Education)			0.83	0.77,0.9	0.87	0.81,0.94
**Participant Education** (Higher Values = Higher Education)					1.16	1.04,1.3
**Smoking Status**					0.95	0.68,1.33
**Change in Self-Reported Health**					0.80	0.69,0.93
**Self-Report of Health Change**					1.74	1.33,2.26
**Chronic Condition Index**					1.08	1.03,1.13
**Change in Functional Limitations**					1.08	0.76,1.52
**BMI**					0.996	0.99,1.0001

	Non-Hispanic White CMV					
	Model 1		Model 2		Model 3	
	Est	CI	Est	CI	Est	CI
**Intercept**	0.25	0.18,0.34	1.87	1.17,2.98	2.48	0.92,6.72
**Experienced Parental Separation**	1.26	1.23,1.3	1.28	1.24,1.32	1.22	1.18,1.26
**Age** (Years)	1.05	1.05,1.05	1.04	1.04,1.04	1.04	1.04,1.04
**Gender (Male vs Female)**	2.21	2.17,2.26	2.10	2.05,2.15	2.12	2.07,2.17
**Parental Education** (Higher Values = Higher Education)		0.,0.	0.63	0.63,0.64	0.67	0.67,0.68
**Participant Education** (Higher Values = Higher Education)					0.79	0.78,0.8
**Smoking Status**					1.12	1.11,1.14
**Change in Self-Reported Health**					1.00	0.99,1.01
**Self-Report of Health Change**					0.90	0.88,0.92
**Chronic Condition Index**					1.03	1.026,1.034
**Change in Functional Limitations**					0.83	0.82,0.84
**BMI**					1.01	1.008,1.011

Panel B. sTNFR

	Non-Hispanic Black sTNFR					
	Model 1		Model 2		Model 3	
	Est	CI	Est	CI	Est	CI
**Intercept**	673.81	637.44,712.26	678.66	605.48,760.68	471.02	413.14,537.02
**Experienced Parental Separation**	1.02	1.,1.03	1.01	0.99,1.03	0.9957	0.9806,1.0109
**Age** (Years)	1.01	1.01,1.01	1.01	1.01,1.01	1.0116	1.0114,1.0119
**Gender (Male vs Female)**	1.01	1.01,1.02	0.99	0.99,1.	0.9732	0.9676,0.9789
**Parental Education** (Higher Values = Higher Education)			0.98	0.98,0.98	0.9994	0.9972,1.0017
**Participant Education** (Higher Values = Higher Education)					0.9771	0.976,0.9782
**Smoking Status**					1.0162	1.013,1.0194
**Change in Self-Reported Health**					0.9892	0.9878,0.9905
**Self-Report of Health Change**					1.0287	1.0244,1.033
**Chronic Condition Index**					1.0810	1.0795,1.0824
**Change in Functional Limitations**					1.0826	1.0789,1.0864
**BMI**					1.0085	1.0079,1.0091

	Hispanic sTNFR					
	Model 1		Model 2		Model 3	
	Est	CI	Est	CI	Est	CI
**Intercept**	896.61	778.53,1032.61	926.66	731.26,1174.28	852.05	538.85,1347.31
**Experienced Parental Separation**	0.98	0.97,0.99	0.98	0.96,0.99	0.95	0.93,0.96
**Age** (Years)	1.01	1.01,1.01	1.01	1.01,1.01	1.01	1.01,1.01
**Gender (Male vs Female)**	0.97	0.95,0.98	0.97	0.95,0.98	0.93	0.92,0.94
**Parental Education** (Higher Values = Higher Education)			0.98	0.97,0.98	1.01	1.01,1.02
**Participant Education** (Higher Values = Higher Education)					0.96	0.95,0.97
**Smoking Status**					1.06	1.06,1.07
**Change in Self-Reported Health**					0.94	0.94,0.95
**Self-Report of Health Change**					1.06	1.04,1.07
**Chronic Condition Index**					1.09	1.09,1.09
**Change in Functional Limitations**					1.04	1.04,1.05
**BMI**					1.00	1.001,1.002

	Other Race sTNFR					
	Model 1		Model 2		Model 3	
	Est	CI	Est	CI	Est	CI
**Intercept**	452.59	406.57,503.83	531.87	461.44,613.06	464.14	309.11,696.91
**Experienced Parental Separation**	1.03	1.01,1.05	1.01	0.99,1.03	0.97	0.95,0.99
**Age** (Years)	1.02	1.02,1.02	1.02	1.02,1.02	1.01	1.01,1.01
**Gender (Male vs Female)**	1.04	1.03,1.05	1.07	1.06,1.07	0.99	0.977,0.995
**Parental Education** (Higher Values = Higher Education)			0.96	0.95,0.96	0.99	0.99,0.996
**Participant Education** (Higher Values = Higher Education)					0.94	0.935,0.947
**Smoking Status**					0.94	0.932,0.945
**Change in Self-Reported Health**					1.00	1.,1.
**Self-Report of Health Change**					1.03	1.02,1.04
**Chronic Condition Index**					1.07	1.07,1.07
**Change in Functional Limitations**					1.09	1.08,1.1
**BMI**					1.01	1.01,1.01

	Non-Hispanic White sTNFR					
	Model 1		Model 2		Model 3	
	Est	CI	Est	CI	Est	CI
**Intercept**	611.73	608.43,615.05	688.88	684.69,693.11	465.42	459.37,471.56
**Experienced Parental Separation**	1.04	1.04,1.05	1.04	1.03,1.04	1.020	1.017, 1.025
**Age** (Years)	1.01	1.01,1.01	1.01	1.01,1.01	1.0129	1.01286, 1.012934
**Gender (Male vs Female)**	1.00	1.,1.	1.00	1.,1.	1.0017	1.001, 1.002
**Parental Education** (Higher Values = Higher Education)			0.97	0.97,0.97	0.9896	0.989, 0.99
**Participant Education** (Higher Values = Higher Education)					0.9617	0.961, 0.962
**Smoking Status**					0.9992	0.999, 1.00
**Change in Self-Reported Health**					0.999361	0.9991, 0.9996
**Self-Report of Health Change**					1.018129	1.017, 1.019
**Chronic Condition Index**					1.05142	1.05114, 1.517
**Change in Functional Limitations**					1.0226	1.022,1.023
**BMI**					1.0126	1.012,1.013

Panel C. IL-6

	Non-Hispanic Black IL-6					
	Model 1		Model 2		Model 3	
	Est	CI	Est	CI	Est	CI
**Intercept**	1.37	1.15,1.62	1.12	0.89,1.42	0.47	0.31,0.72
**Experienced Parental Separation**	0.93	0.92,0.95	0.95	0.94,0.97	0.93	0.92,0.95
**Age** (Years)	1.01	1.01,1.02	1.02	1.02,1.02	1.022	1.0216,1.023
**Gender (Male vs Female)**	1.17	1.15,1.18	1.15	1.13,1.17	1.14	1.12,1.15
**Parental Education** (Higher Values = Higher Education)			0.96	0.95,0.96	0.96	0.95,0.96
**Participant Education** (Higher Values = Higher Education)					1.04	1.03,1.04
**Smoking Status**					1.13	1.12,1.14
**Change in Self-Reported Health**					1.08	1.08,1.09
**Self-Report of Health Change**					0.98	0.97,0.99
**Chronic Condition Index**					1.04	1.03,1.04
**Change in Functional Limitations**					0.99	0.9883,0.9959
**BMI**					1.016	1.01,1.02

	Hispanic IL-6					
	Model 1		Model 2		Model 3	
	Est	CI	Est	CI	Est	CI
**Intercept**	0.37	0.2,0.7	0.26	0.13,0.55	0.15	0.04,0.64
**Experienced Parental Separation**	0.90	0.87,0.94	0.91	0.87,0.94	0.88	0.85,0.91
**Age** (Years)	1.03	1.03,1.03	1.04	1.04,1.04	1.04	1.03,1.04
**Gender (Male vs Female)**	1.19	1.16,1.23	1.22	1.18,1.26	1.15	1.11,1.19
**Parental Education** (Higher Values = Higher Education)			1.05	1.03,1.06	1.09	1.08,1.11
**Participant Education** (Higher Values = Higher Education)					0.91	0.9,0.91
**Smoking Status**					1.10	1.06,1.14
**Change in Self-Reported Health**					1.03	1.02,1.05
**Self-Report of Health Change**					1.09	1.06,1.11
**Chronic Condition Index**					1.04	1.04,1.05
**Change in Functional Limitations**					1.06	1.04,1.08
**BMI**					1.02	1.02,1.02

	Other Race IL-6					
	Model 1		Model 2		Model 3	
	Est	CI	Est	CI	Est	CI
**Intercept**	1.25	0.63,2.48	2.21	0.99,4.92	0.56	0.11,2.82
**Experienced Parental Separation**	0.83	0.8,0.87	0.85	0.8,0.89	0.89	0.87,0.92
**Age** (Years)	1.01	1.01,1.02	1.01	1.01,1.01	1.00	1.,1.01
**Gender (Male vs Female)**	1.14	1.07,1.22	1.11	1.02,1.21	0.90	0.87,0.93
**Parental Education** (Higher Values = Higher Education)			0.95	0.93,0.96	1.11	1.07,1.15
**Participant Education** (Higher Values = Higher Education)					0.76	0.72,0.8
**Smoking Status**					0.89	0.86,0.91
**Change in Self-Reported Health**					0.87	0.83,0.9
**Self-Report of Health Change**					1.23	1.11,1.37
**Chronic Condition Index**					1.08	1.07,1.08
**Change in Functional Limitations**					1.04	1.01,1.08
**BMI**					1.06	1.06,1.06

	Non-Hispanic White IL-6					
	Model 1		Model 2		Model 3	
	Est	CI	Est	CI	Est	CI
**Intercept**	1.40	1.37,1.44	1.66	1.59,1.73	0.65	0.6,0.7
**Experienced Parental Separation**	1.067	1.06,1.08	1.069	1.06,1.08	1.033	1.024,1.042
**Age** (Years)	1.017	1.017,1.018	1.017	1.016,1.017	1.016	1.016,1.017
**Gender (Male vs Female)**	0.920	0.918,0.922	0.918	0.915,0.92	0.931	0.929,0.933
**Parental Education** (Higher Values = Higher Education)			0.945	0.944,0.945	0.981	0.98,0.982
**Participant Education** (Higher Values = Higher Education)					0.950	0.949,0.951
**Smoking Status**					1.075	1.074,1.077
**Change in Self-Reported Health**					1.009	1.008,1.009
**Self-Report of Health Change**					0.995	0.993,0.997
**Chronic Condition Index**					1.071	1.07,1.072
**Change in Functional Limitations**					1.042	1.04,1.045
**BMI**					1.028	1.028,1.029

Panel D. CRP

	Non-Hispanic Black CRP					
	Model 1		Model 2		Model 3	
	Est	CI	Est	CI	Est	CI
**Intercept**	1.64	1.,2.71	2.08	1.15,3.76	0.23	0.09,0.58
**Experienced Parental Separation**	0.96	0.92,0.99	0.95	0.91,0.98	0.93	0.9,0.97
**Age** (Years)	1.000	0.9987,1.0013	0.999	0.9976,0.9998	1.006	1.005,1.0069
**Gender (Male vs Female)**	1.43	1.4,1.47	1.45	1.41,1.49	1.34	1.3,1.37
**Parental Education** (Higher Values = Higher Education)			0.87	0.86,0.88	0.89	0.88,0.9
**Participant Education** (Higher Values = Higher Education)					1.02	1.01,1.03
**Smoking Status**					1.16	1.15,1.18
**Change in Self-Reported Health**					1.06	1.06,1.07
**Self-Report of Health Change**					0.97	0.95,0.99
**Chronic Condition Index**					1.09	1.08,1.09
**Change in Functional Limitations**					1.08	1.07,1.09
**BMI**					1.05	1.05,1.05

	Hispanic CRP					
	Model 1		Model 2		Model 3	
	Est	CI	Est	CI	Est	CI
**Intercept**	1.00	0.5,1.98	0.75	0.34,1.67	0.11	0.02,0.59
**Experienced Parental Separation**	0.90	0.86,0.94	0.96	0.91,1.	0.90	0.86,0.94
**Age** (Years)	1.01	1.01,1.01	1.01	1.01,1.01	1.01	1.01,1.02
**Gender (Male vs Female)**	1.36	1.3,1.43	1.42	1.35,1.5	1.28	1.23,1.34
**Parental Education** (Higher Values = Higher Education)			1.05	1.03,1.07	1.12	1.09,1.14
**Participant Education** (Higher Values = Higher Education)					0.90	0.89,0.91
**Smoking Status**					1.10	1.07,1.14
**Change in Self-Reported Health**					1.02	1.004,1.032
**Self-Report of Health Change**					1.19	1.15,1.22
**Chronic Condition Index**					0.98	0.97,0.99
**Change in Functional Limitations**					1.14	1.12,1.15
**BMI**					1.048	1.046,1.05

	Other Race CRP					
	Model 1		Model 2		Model 3	
	Est	CI	Est	CI	Est	CI
**Intercept**	2.83	0.59,13.66	7.97	1.14,55.58	1.37	0.04,49.76
**Experienced Parental Separation**	0.98	0.94,1.03	1.04	0.98,1.09	1.00	0.96,1.05
**Age** (Years)	0.996	0.994,0.997	0.99	0.99,0.99	0.995	0.994,0.996
**Gender (Male vs Female)**	1.03	0.97,1.097	0.94	0.88,1.01	0.86	0.803,0.915
**Parental Education** (Higher Values = Higher Education)			0.86	0.85,0.87	0.90	0.885,0.907
**Participant Education** (Higher Values = Higher Education)					1.11	1.069,1.152
**Smoking Status**					0.97	0.942,1.004
**Change in Self-Reported Health**					1.21	1.191,1.225
**Self-Report of Health Change**					0.79	0.762,0.826
**Chronic Condition Index**					1.09	1.077,1.1
**Change in Functional Limitations**					1.28	1.22,1.35
**BMI**					1.05	1.05,1.06

	Non-Hispanic White CRP					
	Model 1		Model 2		Model 3	
	Est	CI	Est	CI	Est	CI
**Intercept**	1.85	1.77,1.93	2.32	2.19,2.46	0.28	0.25,0.31
**Experienced Parental Separation**	1.074	1.063,1.085	1.078	1.066,1.089	1.032	1.022,1.043
**Age** (Years)	1.000	0.999,1.	0.998	0.998,0.998	1.001	1.001,1.001
**Gender (Male vs Female)**	1.170	1.166,1.174	1.174	1.17,1.178	1.204	1.201,1.208
**Parental Education** (Higher Values = Higher Education)			0.934	0.933,0.935	0.984	0.983,0.986
**Participant Education** (Higher Values = Higher Education)					0.938	0.936,0.94
**Smoking Status**					1.121	1.119,1.124
**Change in Self-Reported Health**					0.975	0.974,0.976
**Self-Report of Health Change**					1.080	1.077,1.083
**Chronic Condition Index**					1.055	1.054,1.057
**Change in Functional Limitations**					1.048	1.046,1.051
**BMI**					1.055	1.054,1.055
