# Supplementary material for: Biological expressions of early life trauma in the immune system of older adults
Source: PLoS One. 2023 Jun 21;18(6):e0286141. doi: 10.1371/journal.pone.0286141 (PMC10284407; doi:10.1371/journal.pone.0286141)
Supplement: S6 Table — (PDF) [file pone.0286141.s010.pdf]

S6 Table. Exponentiated regression coefficients estimating the association between experiencing parental/caregiver loss and parental separation before the age of 16 years and CMV, sTNF, IL-6, and CRP stratified by race/ethnicity. **Model 1** controls for age, gender, and parental education. **Model 2** controls for age, sex, parental education, and state of birth of the respondent. These models were carried out on the non-imputed study sample who were not missing data on state of birth.

	**Non-Hispanic Whites**				**Non-Hispanic Blacks**				**Hispanics**				**Other Race/Ethnicity**			
	**Model 1**		**Model 2**		**Model 1**		**Model 2**		**Model 1**		**Model 2**		**Model 1**		**Model 2**	
	**Est**	**CI**	**Est**	**CI**	**Est**	**CI**	**Est**	**CI**	**Est**	**CI**	**Est**	**CI**	**Est**	**CI**	**Est**	**CI**
**A. CMV**																
Experienced Parental/Caregiver Loss	1.08	0.82,1.42	1.03	0.78,1.36	1.52	1.01,2.28	1.41	0.3,6.63	1.00	0.67,1.5	0.99	0.53,1.86	0.55	0.21,1.44	0.51	0.28,.91
Experienced Parental Separation	1.42	1.11,1.83	1.32	1.04,1.68	1.67	1.14,2.45	1.58	1.09,2.3	1.55	1.07,2.25	1.44	1.2,1.73	1.68	0.75,3.76	0.85	0.37,1.96
**B. IL-6**																
Experienced Parental/Caregiver Loss	1.15	1.06,1.25	1.16	1.08,1.25	0.96	0.85,1.07	0.96	0.84,1.1	1.00	0.84,1.19	1.02	0.94,1.12	1.15	0.77,1.7	1.26	0.96,1.64
Experienced Parental Separation	1.08	1.01,1.16	1.10	1.01,1.19	0.98	0.87,1.11	1.00	0.86,1.15	1.00	0.85,1.17	1.01	0.88,1.16	0.78	0.54,1.11	0.61	0.37,1.01
**C. CRP**																
Experienced Parental/Caregiver Loss	1.18	1.08,1.3	1.20	1.09,1.31	0.91	0.74,1.13	1.01	0.85,1.19	0.99	0.81,1.21	1.03	0.92,1.14	0.97	0.59,1.61	1.05	0.82,1.35
Experienced Parental Separation	1.09	1.,1.18	1.09	1.,1.2	0.95	0.77,1.18	0.98	0.83,1.16	0.93	0.75,1.15	0.92	0.83,1.02	0.98	0.66,1.46	1.25	0.83,1.87
**D. sTNFR**																
Experienced Parental/Caregiver Loss	1.06	1.03,1.1	1.06	1.03,1.1	0.99	0.9,1.09	0.97	0.9,1.04	0.96	0.87,1.05	0.97	0.93,1.02	1.01	0.89,1.16	0.98	0.87,1.1
Experienced Parental Separation	1.00	0.97,1.03	1.00	0.98,1.03	0.98	0.89,1.07	0.98	0.9,1.08	0.95	0.87,1.05	0.97	0.91,1.03	0.96	0.83,1.11	0.90	0.77,1.06

Model 1 controls for age, sex, and parental education. **Model 2** controls for age, sex, parental education, and state of birth of the respondent.
